# Base and Prime Editing for Inherited Retinal Diseases: Delivery Platforms, Safety, Efficacy, and Translational Perspectives

**DOI:** 10.3390/pharmaceutics17111405

**Published:** 2025-10-30

**Authors:** Haoliang Zhang, Yuxuan Li, Jiajie Li, Xiaosa Li, Tong Li

**Affiliations:** 1 Department of Ophthalmology, Shanghai General Hospital, Shanghai Jiao Tong University School of Medicine, Shanghai 200080, China; dasein@sjtu.edu.cn; 2 National Clinical Research Center for Ophthalmic Diseases, Shanghai 200080, China; 3 School of Medicine, Shanghai Jiao Tong University, Shanghai 200025, China; liyuxuan608@sjtu.edu.cn (Y.L.); lijiajie699604@sjtu.edu.cn (J.L.); 4 Shanghai Engineering Center for Visual Science and Photomedicine, Shanghai 200080, China; 5 Shanghai Key Laboratory of Fundus Diseases, Shanghai 200080, China; 6 Shanghai Gene Therapy Clinical Research Center, Shanghai 200080, China

**Keywords:** inherited retinal diseases, gene therapy, gene editing, base editing, prime editing, gene delivery system, clinical translation challenges, AI-driven system design

## Abstract

Inherited retinal diseases (IRDs) are a clinically and genetically heterogeneous spectrum of disorders that lead to progressive and irreversible vision loss. Gene therapy is the most promising emerging treatment for IRDs. While gene augmentation strategies have demonstrated clinical benefit and results within the first approved ocular gene therapy, their application is restricted by adeno-associated virus (AAV) packaging capacity and limited efficacy for dominant mutations. Recent breakthroughs in precision genome editing, particularly base editing (BE) and prime editing (PE), have provided alternatives capable of directly correcting pathogenic variants. BE enables targeted single-nucleotide conversions, whereas PE further allows for precise insertions and deletions, both circumventing the double-strand DNA cleavage or repair processes typically induced by conventional CRISPR–Cas editing systems, thereby offering advantages in post-mitotic retinal cells. Preclinical investigations across murine and non-human primate models have demonstrated the feasibility, molecular accuracy, and preliminary safety profiles of these platforms in targeting IRD-associated mutations. However, critical challenges remain before clinical application can be realized, including limited editing efficiency in photoreceptors, interspecies variability in therapeutic response, potential risks of off-target effects, and barriers in large-scale vector manufacturing. Moreover, the delivery of genome editors to the outer retina remains suboptimal, prompting intensive efforts in capsid engineering and the development of non-viral delivery systems. This review synthesizes the current progress in BE and PE optimization, highlights innovations in delivery platforms that encompass viral and emerging non-viral systems and summarizes the major barriers to clinical translation. We further discuss AI-driven strategies for the rational design of BE/PE systems, thereby outlining their future potential and perspectives in the treatment of IRDs.

## 1. Introduction

Inherited retinal diseases (IRDs), including retinitis pigmentosa (RP), Leber’s congenital amaurosis (LCA), Stargardt disease, cone-rod dystrophy (CORD), X-linked retinoschisis (XLRS), and choroideremia, represent a group of progressive, often blinding disorders driven by retinal gene mutations, with marked genetic heterogeneity. To date, 367 loci, of which 338 are genes, have been identified [1]. Their prevalence is estimated at ~1 in 3450 individuals globally, with approximately 5.5 million people worldwide affected [2]. These defects primarily affect photoreceptors (rod cells and cone cells) or the retinal pigment epithelium [2], causing irreversible visual impairment like night blindness, central vision loss or color vision impairment or narrowed field of vision. For most IRDs, no disease-modifying treatments are available, and management is limited to supportive measures like visual aids [3]. Conventional pharmacotherapy and surgical interventions do not address the underlying genetic defects; however, there are emerging approaches under active investigation which aim to provide novel therapeutic strategies. However, stem cell therapies lack consistent efficacy and still face unmet challenges in terms of in-cell sourcing and safety [4]. Retinal prostheses only restore basic light perception. And complement-targeting drugs and neurotrophic factors show limited clinical value [5]. Among these, gene therapy stands out as the most promising and widely studied approach with core strategies, such as gene augmentation, gene silencing, and gene editing.

The accessibility, low-dose adaptability, and blood-retinal barrier-mediated immune privilege [6] make eyes ideal for gene therapy. As of 2024, more than 60 IRD gene therapy trials are active globally, but most rely on adeno-associated virus (AAV)-based delivery [7]. A pivotal milestone is the first in vivo gene therapy for genetic disorders approved by the FDA called LUXTURNA. It is an AAV-based augmentation therapy for *RPE65*-linked LCA which that has improved vision in over 400 global patients by 2025 [8,9,10]. Yet, LUXTURNA has limitations: these include temporary benefits due to progressive photoreceptor loss, AAV’s small cargo capacity, immunogenicity, and the inability to re-dose [11]. Despite this, gene augmentation remains one of the most clinically advanced gene therapy strategies for IRDs, though it only treats autosomal recessive and haplo-insufficient IRDs [12,13].

Gene silencing strategies like RNAi and ASOs requires repeated dosing and do not permanently correct mutations [14,15]. Optogenetics faces exogenous light and expression challenges [16]. The research gap surrounding these issues drove gene editing development for a broader IRD spectrum, including those types with dominant-negative mutations. CRISPR/Cas9, the first widely used editing tool [17], now have been used in EDIT-101 trial for *CEP290*-LCA [18] In the most recent 2024–2025 update for the EDIT-101 trial, all subjects demonstrated improvement in visual function, with no reports of adverse events and first pediatric dosing [19]. Nevertheless, CRISPR/Cas9 induces double-strand breaks (DSBs) but showed limitations in terms of low homology-directed repair (HDR) efficiency, uncontrolled indels [20,21], large deletions [22,23], chromosomal translocations [21], DNA breaks [24], and other genomic abnormalities [25]. In addition to Cas9, other CRISPR nucleases such as Cas12a (Cpf1) have been explored for genome editing. Cas12a recognizes T-rich PAM sequences and introduces staggered DNA cuts, enabling multiplexed editing and potentially higher precision in some contexts. However, its lower activity in mammalian cells has so far restricted its application in IRDs.

Base editors (BE, for single-nucleotide corrections [26]) and prime editors (PE, targeting ~89% of pathogenic variants [27,28]) can circumvent DSBs risks and reduce the frequency of indels with higher safety and precision. In addition, no exogenous DNA template for PEs is required [29]. However, their clinical translation for IRDs still faces hurdles, such as suboptimal photoreceptor delivery, vector immunogenicity, and scalable production gaps. Artificial intelligence (AI) is emerging to mitigate these issues, aiding editor design, efficiency/off-target prediction, and personalized therapy for heterogeneous IRDs [30].

This review outlines advances in base and prime editing for IRDs, focusing on delivery optimization and preclinical-to-clinical transitions. By highlighting progress, solutions, and unresolved challenges, it aims to guide technical refinement and accelerate translation to safe, effective IRD treatments.

## 2. Technical Fundamentals and Optimization

### 2.1. Classification and Mechanisms of Base and Prime Editors

Base editors and prime editors, both derived from CRISPR Cas9, enable precise genome editing in post-mitotic retinal cells (non-dividing photoreceptors and RPE cells) without generating DSBs, thereby avoiding the high risk of indels, large deletions, or chromosomal abnormalities that can occur in these cells due to their limited DNA repair capacity. This property distinguishes them from conventional gene augmentation approaches, which deliver a functional gene copy but do not correct the endogenous mutation.

**Figure 1:**
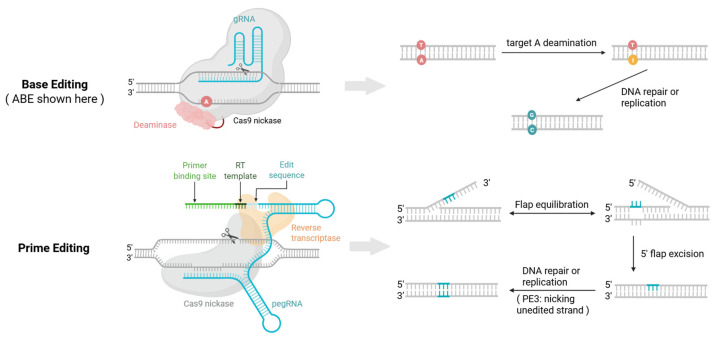
Mechanisms of base editing and prime editing. Base editing (A→G, converting A•T to G•C as an example): The guide RNA (gRNA) directs the base editor complex to the target site. The binding of nCas9 (or dCas9) opens a single-stranded DNA “bubble,” exposing a window of bases on the non-complementary strand. Within this window, an adenosine deaminase converts the target ‘A’ to inosine (I). This process may also unintentionally modify other susceptible bases within the activity window, leading to bystander edits. During DNA replication/repair, ‘I’ is interpreted as ‘G’ and pairs with ‘C’, leading to the synthesis of a ‘C’ on the opposite strand. Nicking the non-edited strand biases repair toward the intended G•C base pair [32]. Prime editing: A prime editing guide RNA (pegRNA) directs the prime editor (nCas9 (H840A) fused to a reverse transcriptase) to the target site. nCas9 introduces a single-strand nick in the protospacer adjacent motif (PAM)-containing DNA strand, exposing a 3′ end. The “primer binding site” (PBS) of the pegRNA anneals to this 3′ end. Crucially, the reverse transcriptase then directly synthesizes a new DNA sequence guided by the “reverse transcription template” (RTT) within the pegRNA, creating a 3′ DNA flap with the desired edit. This template-driven mechanism precisely installs the intended change without an activity window, thereby avoiding the bystander mutations associated with base editing. Flap resolution and DNA repair incorporate the new sequence into the genome. In PE3, an additional nick on the non-edited strand can further increase installation efficiency [33]. ((Created in BioRender: Zhang, H. (2025). https://BioRender.com/t0kvnl2).

Base editors achieve targeted single nucleotide conversions through deamination reactions. The first reported type, cytosine base editors (CBEs), combines cytidine deaminase with a catalytically impaired Cas enzyme and uracil glycosylase inhibitor to convert cytosine guanine base pairs to thymine adenine [26]. Adenine base editors (ABEs) followed, using evolved deoxyadenosine deaminase to turn adenine thymine pairs into guanine cytosine (Figure 1) [31].

These early editors could only reverse around 61% of pathogenic point mutations; so, hundreds of BEs with varied properties have since emerged and can be classified into nuclear DNA base editors, mitochondrial DNA base editors, and RNA base editors. Functionally, in addition to CBEs [26,34] and ABEs [31], C-to-G base editors (CGBEs) and adenine transversion base editors (AYBEs) enable base transversions [35,36]. Dual base editors capable of simultaneously editing adenine and cytosine, such as STEME [37], ACBE [38], and AGBE [39], have also been developed. Based on the editing substrate, in addition to the aforementioned nuclear DNA base editors, RNA base editors (such as REPAIR [40] and RESCUE [40]) and mitochondrial base editors (mitoBEs), such as DdCBE [41] and TALED [42] have also been developed.

BEs have been applied to correct pathogenic point mutations in IRDs [43]. However, they cannot handle all single-nucleotide changes or mediate insertions and deletions (Table 1).

Prime editors address these limitations by fusing a reverse transcriptase to a Cas9 nickase. Their guide RNA called pegRNA includes a primer binding site to direct binding and a reverse transcriptase template encoding the desired edit [28]. The pegRNA first binds the target DNA sequence. The editor then nicks the non-target strand allowing the primer binding site on the pegRNA to attach and start reverse transcription, which generates a single stranded 3′ flap with the edit. This edited flap competes with the unedited 5′ flap for integration. Endonucleases remove the 5′ flap while the 3′ flap anneals to the complementary strand. DNA polymerase extends the sequence using the edited strand as a template and DNA ligase seals the break. The DNA repair system then uses the modified strand to fix the other strand stabilizing the edit [28]. Prime editors can achieve all 12 possible base to base conversions plus small insertions and deletions covering around 89% of known pathogenic mutations, making them a more versatile tool for IRD treatment (Figure 1) [28].

### 2.2. Optimization Strategies for Base Editors

Base editors show promise for gene therapy but face technical bottlenecks in clinical translation. The first-generation BE1 enabled precise single-base substitution, yet had low efficiency, needing an extra DNA replication cycle to introduce edits into target strands [26]. Later versions like BE3, BE4 and BEmax boosted efficiency but caused non-specific nicks on non-target DNA—triggering repair processes that risk off-target edits and potential toxicity or carcinogenicity [44]. They also struggled with narrow editing windows (limiting therapeutic use) and bystander editing because prolonged Cas9-DNA binding leads to the unintended deamination of bases in the editing window, modifying nucleotides adjacent to the target and causing unpredictable phenotypes [45].

Early cytosine and adenine base editors also had significant RNA off-target activity, inducing guide RNA-independent edits across the transcriptome and even self-editing their own transcripts—disrupting editor coding sequences [46]. Researchers addressed this with SECURE-BE3 variants to suppress unwanted RNA editing while keeping high DNA on-target efficiency [46]. Cytidine deaminases from different sources varied in RNA off-target risk: human APOBEC3A-based CBEs caused heavy RNA editing, while improved versions like CBE6, CBE7 and Target AID had lower activity, offering solutions for RNA-related off-target issues [46].

Codon optimization and nuclear localization signal design are key to boosting DNA editing efficiency. Cell protein expression depends on codon usage; so, matching optimization to hosts and scenarios matters [29]. For example, adding a bipartite nuclear localization signal to BE4’s N and C termini raised average efficiency by 1.3-fold [47]. Then further codon re-optimization with GenScript turned this modified BE4 into the more efficient BE4max [47]. The same strategy for ABE7.10 created ABE7.10max, which enhanced editing and slightly increased indel formation [47]. Codon re-engineering made a next-generation BE3, which successfully resulted in up to 30-fold higher efficiency across mammalian cells and tissues, as well as low indel rates [48]. In addition, later studies added a nuclear localization signal and FLAG tag to this BE3-created FNLS-BE3, thus doubling efficiency [48]. These studies confirm that codon and nuclear localization signal tweaks effectively improve BE performance. QBEmax fuses an engineered deaminase with a Cas9 nickase variant. It controls whole-genome off-target effects, with an indel rate of only 0.53 ± 0.21% at the *PDE6B* site, and achieves 27.8 ± 4.1% targeted editing in macaques—providing a high-precision tool for IRD treatment [49].

For therapeutic targets sensitive to bystander effects, base editors with narrow editing windows, expanded PAM compatibility, and enhanced specificity are preferred. These improvements aim to precisely achieve intended substitutions while minimizing unintended edits, thereby increasing both the safety and reliability of base editors for clinical applications.

Studies to date suggest that when bystander edits simply cannot be tolerated at a given target, a base editor with a broad editing window might not be your best option. Going forward, the field is trending toward developing base editors that recognize a wider variety of PAMs, feature much narrower editing windows, and boast higher specificity. That way, you keep unintended “bystander” changes to a minimum while still nailing the exact base swap you want. As these tweaks add up, base editors should find a better sweet spot between efficiency and precision, making them more dependable and safer for clinical applications.

### 2.3. Optimization Strategies for Prime Editors

Prime editors enable broader gene edits than base editors and have lower off-target mutation rates in cellular models and at targeted sites tested in vivo [50,51]. Yet, they have clear limitations.

Structurally they rely on Cas9 PAM sequences restricting the target site range. PegRNA length impacts editing efficiency with effects varying across sites and models [50]. PE3 needs a second nicking site in order to add design complexity and mutation risk Functionally they show low overall efficiency and produce byproducts like insertion-deletion mutations. Such inconsistent outcomes remain a key hurdle for therapeutic translation.

During development, the first-generation PE1 achieved edits via single DNA breaks but with low efficiency. Later iterations improved efficiency greatly. First, PE2 increased efficiency through codon optimization [28]. Next, PE3 added a second break to boost efficiency, despite indel rates rising [28]. And then PEmax optimized activity and nuclear localization via amino acid substitutions and nuclear localization signals [52]. Further optimizations include nuclease PE for hard-to-edit cells and dual PE systems for larger repair fragment insertions [53,54]. Phage-assisted continuous evolution yielded PE6 with 183-fold higher activity than the original system [55].

For precision, the vPE system adjusts Cas9 nicking sites. It maintains high editing efficiency while cutting indel frequency by up to 60-fold, thus achieving an editing-to-indel ratio of 465:1 to improve accuracy [56]. The circular RNA-mediated ciPE targets regions upstream of cut sites in human cells expanding editable genomic range. Its editing efficiency ranges from 0.1% to 24.7% and increased to a range of 2.7% to 55.4% with Rep-X helicase. It has high purity with minimal indels and low off-target effects and performed well in editing *RPE65* [57].

To address large-fragment integration challenges, Pandey et al. developed the PASSIGE system, which combines prime editing with the Bxb1 recombinase. Through phage-assisted evolution (PACE and PANCE), they generated evolved variants (evoBxb1, eeBxb1) that markedly improved integration efficiency (up to 9.1-fold and 16-fold higher than the PASTE system) and enabled precise insertion of fragments exceeding 10 kb while maintaining low off-target activity [58,59]. Despite these advances, several issues limit PASSIGE’s immediate application in IRDs. The potential immunogenicity of recombinase proteins, particularly with repeat dosing or long-term expression, remains untested. In addition, AAV vector size constraints hinder efficient co-delivery of the recombinase, prime editor, and large donor fragments to retinal cells. Furthermore, pseudo-attB recognition may cause rare off-target recombination events, and in vivo validation in retinal models has not yet been reported. Although current studies report minimal off-target editing effects [58], further validation studies are necessary for safe translation of PASSIGE in IRD therapy.

The length of the prime editor (6.3 kb) exceeds the packaging capacity of AAV. Dual-AAV split strategies improve delivery but cut efficiency. A recent study developed a modular SunTag-PE system, splitting the PE effector into GCN4-nCas9 and a single-chain variable fragment tethered reverse transcriptase. The setup with one GCN4 at the nCas9 N-terminus had optimal efficiency, matching traditional fused PE in PE2 and PE3 forms, and outperforming other split strategies like sPE and MS2-PE without more indels. The dual AAV delivery of SunTag-ePE3 corrected pathogenic mutations in an HBB mutant cell line, providing an efficient split strategy for clinical translation [60].

### 2.4. Therapeutic Potential of Base and Prime Editors Targeting IRDs

Base editing and prime editing technologies have paved the way for unprecedented possibilities for the treatment of IRDs, offering broader applicability and curative potential for IRD patients with complex genetic backgrounds and diverse mutation types.

Studies indicate that over 55% of *RHO*-associated RP variants are amenable to treatment with BE or PE technologies, a proportion likely to increase further with the application of Cas9 variants with relaxed PAM requirements such as SpRY-Cas9 and Cas9-NG [61]. Preclinical studies show that even with overall DNA editing rates below 15%, significant functional phenotypic recovery can be induced [43], highlighting the retina’s suitability as a target organ. Retinal cells are highly differentiated and non-dividing, stably retaining gene vectors, while the retina’s blood-retinal barrier and established local delivery routes—intravitreal or subretinal injection—enable efficient vector delivery and limit systemic immune responses [62,63]. Moreover, ocular tissues are easily accessible for monitoring, supported by abundant animal models, well-defined monogenic mechanisms, and a regulatory precedent in the form of FDA-approved LCA2 gene therapy, all of which favor the clinical translation of BE and PE in IRDs.

To date, BE has been used to develop treatments for several IRDs, including two recessive RP subtypes caused by *PDE6B* c.1678 C > T and *RPE65* exon 3 T > C mutations. Cas9 variants with relaxed PAM sequences like SpRY-Cas9 and Cas9-NG were used for targeting. In P14 *rd10* mice, the intraretinal injection of SpRY-Cas9 or Cas9-NG-mediated BE achieved an approximately 13% specific mutation correction in the retina [64,65]. Su et al. used NG-ABE8e and detected an average DNA editing efficiency of ~30% at 12 weeks post-treatment when the outer nuclear layer contained 2–3 cone nuclei per row. In both cases, optical coherence tomography (OCT) measured the outer nuclear layer’s thickness and electroretinography detected retinal function that were maintained or restored, revealing therapeutic-level gene editing [66].

However, NG-ABE8e appeared more effective in vivo at the *PDE6B* site than SpRY-ABE8e though more comprehensive SpRY analysis is needed. Others used NG-ABEmax to target *RPE65* and observed relatively low yet variable editing levels post-subretinal injection across research groups with no significant effect [67,68]. For this mutation, protein restoration is key to phenotypic recovery. In both cases, over 80% of post-treatment cDNA reads came from the corrected allele, indicating protein restoration and leading to significant phenotypic recovery.

BE also plays a role in RNA gene editing. Fry et al. combined RNA-targeting Cas13 with adenosine deaminase acting on RNA to repair *USH2A* mutations. They successfully corrected the common human mutation c.11864 G > A and its murine counterpart c.11840 G > A with in vitro correction rates of up to 80%. The subretinal injection of AAV8-Cas13-ADAR in *USH2A* W3947X/W3947X mice achieved maximum editing efficiency of 2.04% ± 0.16 with ~30% retinal transduction. This editing increased Usherin expression, resulting in potential for Cas13-ADAR therapeutics [69].

Prime editing has also been used for targeted IRD therapy. SpRY-PE2 delivered via a dual-AAV system achieved over 75% editing efficiency in the transduced cells of *rd10* mice. Phenotypic analysis showed significant improvements in outer nuclear layer thickness rod photoreceptor stability and retinal function compared to untreated controls. In contrast, *rd12* mice treated with SpCas9-PE3 showed relatively lower editing levels with a maximum average rate of only 11.4 ± 2.3% [70].

**Figure 2:**
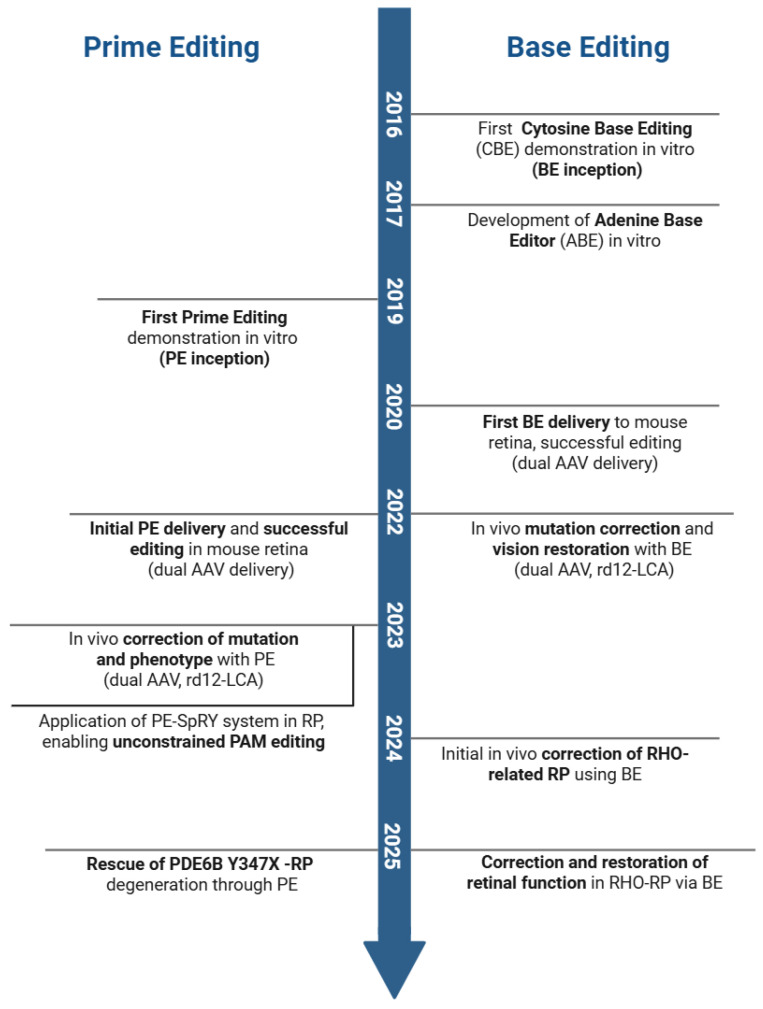
Key milestones of base and prime editing for inherited retinal diseases (IRDs). Seminal publications corresponding to the events on the timeline are as follows: First demonstration of Cytosine Base Editing (CBE) in vitro (BE inception) [26]; Development of Adenine Base Editor (ABE) in vitro [31]; First BE delivery to mouse retina, successful editing (dual AAV delivery) [73]; In vivo mutation correction and vision restoration with BE (dual AAV, *rd12*-LCA) [67]; Initial in vivo correction of RHO-related RP using BE [74]; Correction and restoration of retinal function in RHO-RP via BE [75]; First Prime Editing demonstration in vitro (PE inception) [28]; Initial PE delivery and successful editing in mouse retina (dual AAV delivery) [76]; In vivo correction of mutation and phenotype with PE (dual AAV, *rd12*-LCA) [77]; Application of PE-SpRY system in RP, enabling unconstrained PAM editing [78]; Rescue of *PDE6B* Y347X mutation-induced RP degeneration through PE [71]. (Created in BioRender: Zhang, H. (2025). https://BioRender.com/t0kvnl2).

For autosomal dominant IRDs such as *RHO* mutations, PE7 fused with the viral La protein, which improved reverse transcriptase template stability, increasing the in vivo editing of the Pro23His mutation from 43% to 68.9% under dual-AAV delivery without increasing inflammation [71]. In addition, it also achieved 91.2% efficiency in mutant allele knockout and 76.3% efficiency in wild-type sequence insertion [71]. Separately, Prime Medicine reported that its dual-AAV prime editing system corrected up to 70% of *RHO* mutations in humanized mouse photoreceptors, with the restoration of retinal structure and function in mouse and retinal explant models [72]. The high editing rates reported here indicate that PE can drive therapeutically meaningful gene correction for *RHO* and other related IRDs (Figure 2).

## 3. Delivery System Innovations

Most current gene-editing clinical trials, including those for BE and PE, use ex vivo editing, where cells are extracted from patients, edited outside the body, and reinfused [79]. This approach is limited to accessible cell types like hematopoietic stem cells [80], whereas ocular cells are less amenable to ex vivo manipulation. The eye’s accessibility, immune-privileged environment, and post-mitotic retinal cells make it well-suited for in vivo delivery [62,63], enabling efficient, localized editing with minimal systemic exposure.

Successful in vivo delivery requires precise targeting of ocular cells and safe delivery of sufficient editors, but delivering macromolecules like base editors remains challenging. A key example is LUXTURNA, the AAV-based gene augmentation therapy for LCA2 that succeeded in 2017, with its translation relying on AAV/retro/lentiviral vector development [11].

Effective in vivo delivery needs gene-editing reagents to overcome intracellular barriers, avoid degradation, and reach the nucleus. Most potent vectors use protein or lipid shells to protect cargo before cellular entry [81], evade immune recognition, and use surface moieties to bind ocular cell receptors [81]. Local injections bypass barriers to access critical cell populations, and vectors often exploit the acidic endosomal environment for escape [81,82].

Delivery systems also need to maximize targeting efficiency and minimize off-target activity that is increased by prolonged base editor expression [83]; so, timely expression control is critical. When selecting vectors for therapeutic BE/PE, factors like payload type (DNA, mRNA, or ribonucleoprotein [RNP]), ocular microenvironment, desired outcomes, and immune compatibility must be considered, alongside evaluations of ocular toxicity, editing/delivery efficiency, payload capacity, and stability.

The type of payload matters significantly. DNA delivery sustains expression but increases off-target/insertion risks [84,85] and only slowly initiates editing. The delivery of mRNA enables faster editing and lower off-target risks via transient expression [85], with instability mitigated by chemical modifications [86]. RNP delivery offers the fastest response and minimal off-target effects [87] for coordinated delivery but is costlier, hindered by Cas protein size, sgRNA charge, and the risk of endotoxins and immune reactions [88].

After decades of research, in vivo delivery systems fall into viral and non-viral categories. Here, we outline preclinical or clinical strategies for vectors like viral vectors, lipid nanoparticles (LNPs), and virus-like particles (VLPs). Since no single method suits all applications, we highlight strengths, weaknesses, advancement opportunities, and how BE/PE synergize with these vectors to accelerate IRD-treating progress.

### 3.1. Viral Vector Delivery Methods

**Figure 3:**
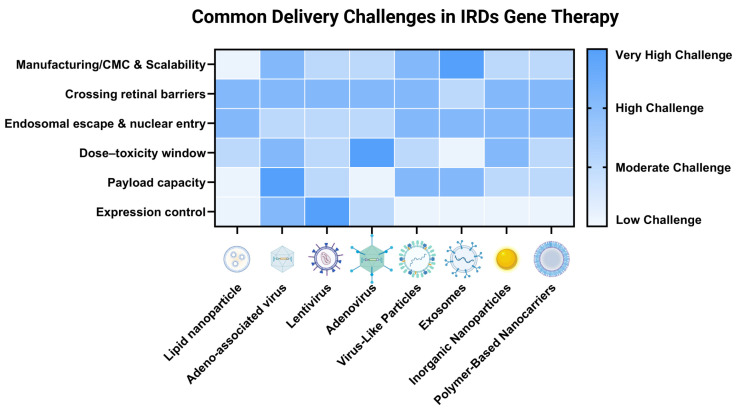
Common delivery systems in gene therapy for IRDs and their shared challenges. The heatmap illustrates the relative significance of six major challenges across eight representative delivery platforms for ocular gene editing. The color intensity corresponds to the estimated magnitude of each challenge, with darker shades indicating a more significant hurdle that currently limits the system’s clinical translation for treating genetic eye diseases. Challenges are qualitatively categorized into four levels: low (lightest shade), moderate, high, and very high (darkest shade). These assessments are based on a synthesis of current literature and represent general trends rather than absolute, quantitative measurements [9,90,91,92,93]. The relative importance of each challenge can vary depending on the specific editor payload (e.g., base editor vs. prime editor), the target retinal cell type, and the route of administration. (Created in BioRender: Zhang, H. (2025). https://BioRender.com/t0kvnl2).

Viral vectors are widely used for delivering genome-editing agents, with over 1000 clinical trials employing them to deliver therapeutic genes or editors [89]. The main types for in vivo genome editing include AAV, lentivirus (LV), and adenovirus (AdV), while others, like Sendai virus and retroviruses, have been explored to a lesser extent (Figure 3, Table 2).

For in vivo genome editing via viral transduction, viral particles first recognize target cell receptors, followed by vector uncoating, cargo transport and release, and transgene transcription and translation. However, viral vectors face several key challenges for ocular base or prime editing. In production, natural AAV variants can meet clinical trial requirements, but broader patient use demands improvements in efficiency and manufacturing processes, as producing high-purity vectors remains complex and labor-intensive. Immunologically, viral capsids can trigger robust innate and adaptive inflammatory responses. Safety-wise, they have limited cargo capacity, risk random genomic integration (which may activate oncogenes), and intraocular vectors might enter the brain via retinal ganglion cell uptake. Additionally, the persistent expression of DNA or genomic RNA increases off-target editing risks and may cause the immune-mediated clearance of edited cells expressing editor proteins [94].

The advantages and disadvantages of viral vectors for in vivo base and prime editing in ocular tissues are discussed below, along with opportunities for future advancements.

#### 3.1.1. AAV

AAV is a non-enveloped virus (25 nm in diameter) with an icosahedral capsid assembled from 60 viral proteins (VP1, VP2, VP3) [95]. It packages a ~5 kb single-stranded DNA genome and naturally transduces both dividing and non-dividing cells, making it suitable for in vivo ocular gene therapy [92,93,94,95,96,97,98,99]. Its value in ocular 6therapeutics, as evidenced by the FDA-approved LUXTURNA (for LCA) and the EDIT-101 clinical trial further supports its potential as a delivery system for base and prime editors.

AAV-mediated delivery offers low immunogenicity, low toxicity, transient gene expression, and high biocompatibility. Advancements in vector engineering have significantly improved its efficiency, sometimes by over 100-fold [100,101,102,103]. In addition, evolution and rational design have expanded its tissue specificity. By using tissue-specific capsids, promoters, or miRNAs, gene-editing cargo expression can be confined to the eye, minimizing off-target editing in non-target tissues.

The first strategy to enhance ocular targeting is leveraging natural serotype tropism. AAV serotypes 2, 5, 6, 7, 8, and 9 and engineered variants like AAV2-7m8 and AAV9-PHP.eB transduce photoreceptors via subretinal injection [104,105]. The high-dose intravitreal injection of certain serotypes targets both photoreceptors and retinal pigment epithelial (RPE) cells, offering a less invasive option for patients with fragile retinas [106,107]. Hung et al. validated safe intravitreal AAV2-Cas9 delivery, reducing YFP expression by 84% in Thy1-YFP mice without functional or morphological damage [108]. Secondly, tissue-specific promoters drive editor expression but are limited by AAV’s packaging capacity. Thirdly, miRNA regulation is more feasible. Xiao et al. integrated tissue-specific miRNA binding sites into the 3′UTR of editor cassettes to suppress activity in non-retinal tissues while maintaining expression in retinal cells [109], and others further combined it with anti-CRISPR (Acr) proteins to block residual non-retinal editing and enhance ocular safety [110,111]. An et al. optimized a dual AAV system for CBE delivery via neuron-specific promoters and miR-183/122 target sequences to reduce off-target expression in non-target tissues [112]. This tissue-specific regulation concept is translatable to IRDs, helping lower editor expression in non-target ocular tissues.

AAV also has notable drawbacks. Post-transduction, its episomal genomes can stably express genetic material for years in retinal cells [96,113], and prolonged editor expression increases off-target risks [32,44]. Pre-existing antibodies against AAV serotypes or SpCas9 may neutralize vectors/editors [114], and pre-existing T-cell responses against SpCas9 or immune reactions in non-human primate eyes raise concerns about edited cell clearance [94,115]. The immunogenicity of deaminase domains in base editors or CBE’s UGI component remains unstudied. Additionally, random AAV genome integration has been reported in mice, non-human primate, human livers, and Cas9-induced DSB sites in other tissues [116,117,118].

To mitigate sustained expression issues, strategies enabling temporal control have been developed. Monteys et al. designed the X^on^ switch, which uses small-molecule-controlled RNA splicing to regulate AAV transgene expression; a compact X^on^ variant is compatible with SaCas9 in single AAV, enabling the temporal control of in vivo editing [119]. Similarly, blue-light-activated base editors (BLBEs) [120] combine photoswitches with DNA deaminases, restricting active editing to periods of blue light exposure to minimize background editing and unintended genomic and transcriptomic changes. Complementing these, the dual-guider CBE system [121] pairs sgRNA with transcription activator-like effector (TALE) elements so that nCas9 and cytosine deaminase co-localize only at the target site, reducing off-target editing caused by sustained deaminase activity at non-specific loci.

However, the most serious constraint is the small packaging capacity (~5 kb) of AAV. And flanked by essential inverted terminal repeats (ITRs), only ~4.7 kb remains for transgenes [98]. Most SpCas9-based BEs/PEs exceed this limit, and additional space for promoters, guide RNAs, and regulatory elements further strains capacity. To overcome this, researchers have developed multiple solutions, which are discussed below.

##### Dual-AAV

To overcome AAV’s packaging limits, gene-editing components are often divided between two vectors, one encoding Cas9 and the other gRNA(s). Co-administered dual AAVs reconstitute full-length editors in co-transduced cells via DNA-, pre-mRNA-, or protein-level mechanisms [122].

Both mRNA trans-splicing and protein trans-splicing enable reconstitution. Multiple labs developed split intein systems for protein trans-splicing, where editors are split into N- and C-terminal fragments (each fused to split inteins), and intein interaction drives seamless splicing into full-length proteins in target cells [123,124]. Split intein-mediated reconstitution achieves ~4.5-fold higher base-editing efficiency across mouse tissues than mRNA trans-splicing [73], as mRNA trans-splicing requires AAV genome concatemerization and ITR-flanked sequence splicing, which can disrupt pre-mRNA [125,126].

PEs are ~1 kb longer than BEs; so, split intein systems are required to package PEs into dual AAVs. Early in situ in vivo studies used dual AAVs for PE delivery, with 14% and 6% editing efficiency at the *DNMT1* site in mouse liver [127,128], but current PE efficiency via dual AAVs remains significantly lower than that of BEs.

Ongoing research focuses on optimizing split intein systems, such as identifying optimal protein split sites for efficient reconstitution [129] and truncating editors to simplify packaging [127] in order to improve in situ editing efficiency and solidify split AAV’s role in delivering large editors [52,130]. Recently, Davis et al. developed v3em PE-AAV system with a strong Cbh promoter, engineered epegRNA, and PEmax modifications and achieved high-efficiency editing in vivo with low off-target effects [131]. Combining this with retina-specific modifications holds promise for PE-based IRD treatment.

##### Single-AAV

While dual-AAV systems enable therapeutic editing in IRD models, their reliance on co-transduction lowers efficiency relative to single-AAV delivery. Single-AAV systems also simplify manufacturing and minimize immunogenicity, making the optimization of gene editor size to fit AAV’s limited capacity a crucial strategy.

Researchers have identified or engineered compact Cas9 variants for single-AAV packaging (Cas9 + gRNAs). Staphylococcus aureus Cas9 (SaCas9, 3.2 kb) is commonly used, as it fits with 1–2 sgRNA expression cassettes. The ongoing EDIT-101 clinical trial employs the subretinal injection of a single AAV vector delivering SaCas9 and two sgRNAs to delete the pathogenic *CEP290* mutation in LCA10 [18].

Other compact Cas9 variants include Nme2Cas9 (3.24 kb, PAM = N4CC) [132], CjCas9 (2.95 kb, PAM = N4RYAC) [133], and SauriCas9 (3.18 kb, PAM = N2GG) [134]. These variants expand the pool of single-AAV-packageable enzymes. For example, Kim et al. used CjCas9 in a single AAV system, delivering it subretinally to an age-related macular degeneration mouse model to knock out VEGF-A, achieving 20% indel rates in retinal and RPE cells and reducing neovascularization [133].

Recently, these small Cas variants were leveraged to develop single-AAV methods for base editor packaging. Davis et al. screened compact Cas orthologs (SaCas9, Nme2Cas9, CjCas9) to generate ABEs, removing WPRE, using bGH polyadenylation signals to optimize elements and construct single AAV vectors (≤5.0 kb with ITRs) integrating ABE and sgRNA cassettes [135]. Zhang et al. built Nme2-ABE8e, optimizing NLS and vector arrangement, and adding an Acr/miRNA-MRE system to enhance editing specificity, delivering it via single AAV to correct pathogenic mutations [136].

Beyond Cas variants, Yi et al. developed deaminase-independent thymine base editor (TBE) supports single-AAV delivery for IRDs. TBE enables T→C/G/A conversions (covering ~70% of T-related pathogenic SNPs in IRDs). Optimized fusion structures with radiation-resistant Deinococcus radiodurans UNG mutant, nickase Cas9 and miniU6 promoters enable “effector + sgRNA” single-cassette design, thus fitting the capacity of AAV [137].

Single-AAV systems have successfully delivered gene editors [79,133,138], but Cas9 variant PAM availability limits utility. Future efforts should focus on developing more compact Cas9 variants with broader targeting ranges for further advancing single-AAV delivery.

#### 3.1.2. LVs

LVs, derived from HIV-1, are replication-incompetent enveloped viruses with ~10 kb cargo capacity, enabling full-length editor delivery [139]. They transduce terminally differentiated cells and allow for tropism tuning via pseudo-typing [140,141].

However, LVs are primarily used ex vivo, as in vivo applications face serious limitations, such as narrower cell targeting than AAV, lower therapeutic efficiency at clinical doses, and genomic integration risks including oncogenesis. Integrase-defective LVs (IDLVs) reduce integration greatly but retain residual frequencies [142]. While LVs delivering co-packaged Cas9 mRNA and gRNA are efficacious and safe [143,144], their relatively large size (80–120 nm) limits intravitreal delivery and necessitates subretinal injection for photoreceptor access, which is further hampered by matrix barriers [145]. To sum up, although subretinally injected LV-ABE corrected *RPE65* mutations in LCA mice with an editing rate of 15.95% [146], the in vivo utility of LVs remains limited [147].

#### 3.1.3. AVs

AVs are non-enveloped (90–100 nm) with 36 kb episomal genomes, offering large payload capacity and non-integrating properties [148]. Historically dominant in gene therapy trials, they enable efficient in vivo editing in tissues like liver (36–58% editing rate with PE2) [128].

Ocular applications face critical barriers. While genetic or chemical capsid modifications enable tropism tuning [149], no ocular-specific designs are validated. Immunogenicity was increased by neutralizing antibodies against Cas9 [150], although T-cell cytotoxicity [151], and innate responses remained prohibitive [152]. “Stealthing” strategies reduce antigenicity [148] but do not eliminate risks. In addition, manufacturing challenges persist [153], resulting in AVs being replaced with AAVs in IRDs.

### 3.2. Nonviral Vector Delivery Methods

While viral vectors are widely used in ocular gene therapy, their viral nature and DNA-based delivery make it extremely challenging to develop an ideal vehicle that is fully non-immunogenic, non-integrating, efficient, safe, and capable of packaging all gene-editing components in one vector [90]. Thus, some teams have shifted to nonviral vectors for mRNA/RNP-based therapeutics.

Compared to viral vectors, nonviral vectors are less likely to trigger immune responses in mammalian cells, have less toxic profiles [154], and are easier to handle and produce. However, they have notable limitations. Transfection efficiency is often suboptimal [155], and tissue targeting needs refinement. Additionally, despite their large packaging capacity for base or prime editor components, larger nonviral particles often struggle with cellular uptake and endosomal escape [156,157]. Additionally, nonviral vectors with base editors (whether delivered as DNA, mRNA, or RNP), need to enter the nucleus to edit genomic DNA; thus, nuclear pore size (<10 nm) represents a significant barrier [158].

As nonviral gene therapies advance toward clinical use for IRDs, safety and ethical concerns emerge. While they reduce immunogenicity, the risk of unintended genetic modifications or adverse effects remain, and off-target effects require rigorous scrutiny. Robust preclinical studies using reliable animal models and long-term clinical trials are essential to defining their ocular safety profiles [159].

To date, nonviral delivery methods for base/prime editors include diverse physical and chemical strategies, each requiring matching to specific editing needs. Among these, LNPs and non-replicating VLPs used in vaccines [160] have gained significant attention, with potential as powerful delivery tools for IRD-targeted base/prime editing. The following sections discuss the strengths, weaknesses, and optimization strategies of various nonviral vectors for IRD treatment, categorized by material origin and structural features (Figure 3, Table 2).

#### 3.2.1. Nonviral Nanoparticle Methods

Nanoparticle delivery vehicles are increasingly used to address viral vector limitations in genome editing, as they can be engineered to optimize cargo accommodation and fine-tuned for immune-compatibility via size, shape, or coating modifications [157].

For gene-editing delivery, key nanoparticle types include LNPs, polymeric nanoparticles, and inorganic nanoparticles. Their compatibility varies with payload type (DNA, mRNA, or protein), as detailed in the following subsections.

##### LNPs

Cationic and ionizable lipids like phosphoethanolamine, 1,2-dioleoyl-sn-glycero-3-phosphoethanolamine (DOPE) and 1,2-dioleoyl-3-trimethylammonium-propane (DOTAP) form compact lipoplexes with negatively charged nucleic acids, protecting them from degradation. What is more, lipid composition, particle size, and the charge of these systems are modifiable [161].

LNPs, clinically mature liposome-derived vectors, comprise four components: ionizable/cationic lipids, helper lipids, PEG-modified lipids, and cholesterol. Ethanol-dissolved lipids mix with buffered nucleic acids to form lipid bilayers that encapsulate cargo, with ionizable lipids that enable endosomal escape, PEG lipids that prevent aggregation, and structural lipids that maintain stability [162]. Adjusting component ratios tailors their pharmacokinetics and tissue tropism [163]. Approved by the FDA for RNA vaccine delivery, LNPs are also preferred carriers for in vivo base editor delivery [164].

For delivery, LNPs conjugate peptides or ligands to bind cell receptors and enter via endocytosis. Then ionizable lipids protonate in acidic endosomes to disrupt membranes, enabling endosomal escape and releasing mRNA/RNP-based base editors into the cytoplasm [165]. Compared to viral vectors, LNPs have lower immunogenicity via synthetically biodegradable components and enable transient editor expression, minimizing off-target risks [43,166].

RNP delivery via LNPs enables efficient editing in extrahepatic tissues like retina, with sufficient efficiency to elicit phenotypic responses in post-mitotic cells [167]. Preclinically, Sun et al. engineered (1-aminoethyl)iminobis[N-(oleicylcysteinyl-1-amino-ethyl)propionamide] (ECO) cationic lipids, with protonatable heads, disulfide linkers, and oleoyl tails to encapsulate *RPE65* pDNA. In addition, the retinamide ligand targeting of IRBP and PEG spacers boosted transfection, sustaining *RPE65* expression in mice and delaying cone degeneration [168]. Jang et al. delivered ABE RNPs using Lipofectamine 2000 LNPs, partially correcting *RPR65* in *rd12* mice and, for the first time, demonstrating the feasibility of LNP-mediated base editing for IRD [167]. With their clinical safety, tunability, and compatibility with base editors, LNPs hold strong potential for use in IRD therapies.

Despite progress, LNPs face critical IRD-specific challenges. Localized delivery struggles with precise retinal cell targeting and high gene expression induction. Retinal barriers like outer photoreceptor segments impede LNP uptake, and co-encapsulating large components complicates endosomal escape. Herrera-Barrera et al. developed eLNPs (incorporating β-caryophyllene) to enhance endosomal escape, achieving 54% in vitro PE efficiency—an insights applicable to retinal editing [169,170]. They also identified retinal receptor-binding peptides that enable cone, rod, and RPE mRNA delivery in non-human primates, although the LNP delivery of BEs/PEs and dose-dependent retinal toxicity remain unconfirmed [171]. Gemini surfactant phospholipid nanoparticles (GL-NPs), modified with integrin/CAP peptides, improve retinal plasmid DNA delivery and may be adapted for ABE-RNP/PE-mRNA encapsulation [172].

Lipid-derived systems like lipid protamine DNA (LPD) complexes—modified with TAT peptides, protamine, and NLS—compress DNA for LCA mouse *RPE65* augmentation [173]. For base/prime editing, replacing protamine with RNP-compatible cationic peptides like polyarginine could preserve compression, while retina-specific ligands may enhance photoreceptor/RPE selectivity. Further advances in sections like retinal targeting are needed for LNPs to accelerate translation.

On the delivery side, (LNPs and their derivative lipid carriers have a lot going for them—proven clinical safety, tunable properties, and compatibility with base editing components. By refining retinal targeting, boosting endosomal escape, and reining in any toxicity, LNP-based systems could realistically pave the way for BE therapies to treat inherited retinal diseases like LCA or retinitis pigmentosa.

##### Inorganic Nanoparticles

Inorganic nanoparticles for retinal gene editing include silica metal organic framework (SMOFs), nanodiamonds, gold nanoparticles (AuNPs), and mesoporous silica nanoparticles (SNPs), with SNPs showing validated potential for base editing in IRDs. They offer safety and scalability advantages over viral vectors but face challenges such as poor biodegradability, in vivo accumulation, and potential off-target or toxicity risks concerns from heavy metal or polymer aggregation [174].

SMOFs modified with all-trans retinoic acid (ATRA) efficiently deliver Cas9/gRNA complexes to RPE cells using short-lived RNA cargo but lack photoreceptor targeting capability [175,176]. Similarly, AuNPs (e.g., CRISPR-Gold) facilitate safe Cas9 RNP delivery, achieving modest HDR efficiency, yet no base editing applications have been reported [177].

SNPs provide high nucleic acid encapsulation efficiency, tunable pore size, and surface functionalization. GSH-responsive silica nanocapsules have been used to deliver cytosine base editors targeting the KCNJ13 W53X mutation, restoring Kir7.1 channel activity in non-dividing RPE cells without disrupting the DNA backbone [178]. This design uses disulfide crosslinkers for controlled degradation and PEGylation to enhance stability and circulation, achieving functional rescue with minimal off-target effects.

Other inorganic platforms, including nanodiamonds and supramolecular hybrids, extend Cas9 delivery to photoreceptors, bipolar, or ganglion cells, but rely on long-lasting DNA cargo and have not yet been applied to BE/PE [179]. Similarly, ATRA-modified SMOF hybrids enable efficient RPE editing via Cas9-sgRNA RNP delivery [176] but have not been applied for base editing. Despite these advances, SNPs require improved photoreceptor targeting, and all inorganic nanoparticles need enhanced biodegradability. The continued optimization of surface chemistry and targeting ligands positions SNPs as a promising non-viral platform for the clinical translation of base editing therapies in IRDs.

##### VLPs

VLPs are non-infectious nanocarriers self-assembled from viral structural proteins (e.g., retroviral Gag/Pol and VSV-G envelope) without viral genomes. They deliver gene-editing reagents such as mRNA or proteins, avoiding host genome integration [180] and reduces off-target risks [180,181]. Retaining viral properties such as endosomal escape and tissue targeting, VLPs accommodate large editors and can be modularly engineered for retinal cell specificity, balancing efficiency and safety for IRD therapy.

Most VLP constructs for mRNA/protein delivery are retrovirus-based such as moloney murine leukemia virus (MLV) and HIV-1, whose spherical 100–200 nm structure and modular tropism (envelope-determined) and packaging (capsid-determined) make them suitable for bulky cargo. VLPs are produced via engineered Gag payload fusion vectors, where producer cells assemble Gag payload with envelope proteins, retaining retroviral transduction efficiency without replicative genomes and enabling flexible mRNA or protein packaging [181,182].

VLPs can encapsulate mRNA or RNP, with distinct loading and release mechanisms, as detailed below.

- mRNA-Packaging VLPs

Packaging base/prime editor mRNAs (with sgRNAs) into VLPs relies on specific molecular recognition motifs. Early retroviral Ψ-based systems, such as MLV VLPs, enabled mRNA encapsulation but were limited to 2–3 RNAs per particle and required mRNA modification to block reverse transcription, restricting cargo capacity [183].

Prel et al. improved VLP mRNA capacity using the MS2apt-MS2cp system, replacing HIV-1 Gag’s ZF2 domain with MS2cp and adding 12 MS2apt to luciferase mRNA’s 3′UTR, enabling 5–6 mRNAs per VLP [184]. Adaptations for Cas9 included Galla et al.’s fusion of 2 MS2cp to MLV Gag’s C-terminus and addition of 2 MS2apt to SpCas9 mRNA/sgRNA, though insufficient sgRNA delivery limited editing efficiency [185]. Lu et al. linked 2 MS2cp to HIV-1 Gag’s ZF2 and added 1 MS2apt to SaCas9 mRNA, co-delivering with IDLV-sgRNA for HEK293T editing, but this approach remained unverified in vivo [186].

Ling et al. applied a similar approach to package SpCas9 mRNA into HIV-1 VLPs with 6 MS2apt on the mRNA 3′UTR, creating all-in-one mLPs co-packaging SpCas9 mRNA and IDLV-sgRNA. Subretinal injection in mice achieved 44% Vegf knockout in RPE cells, while intracorneal injection targeting two proteins validated ocular applicability [143].

Challenges remained because unmodified sgRNAs degrade before Cas9 translation, and IDLV-sgRNA persists as extrachromosomal DNA with residual integration risk [142,187]. Viral scaffold-derived VLPs also require evaluation for immunogenicity. To address these issues, humanized VLPs have been developed. Segel et al. reprogrammed mammalian PEG10, a Gag homolog, to package editor mRNAs via its UTRs (SEND system), enabling cell-specific delivery through targeting fusion proteins. MmPEG10-VLPs produced efficient transient retinal expression with minimal immunogenicity after subretinal injection, and in *rd12* mice, SEND-VLP-delivered ABEs achieved 1.8-fold higher on-target editing than AAVs with minimal off-target effects [92,188], highlighting VLP potential for IRD base editing.

- RNP-Packaged VLPs

The development of RNP-packaged VLPs for IRD base and prime editing has progressed from early HIV-1- and MLV-based designs, which lacked pre-assembled RNPs, faced Cas9 dose limitations, or were untested in retinal cells [189,190], to optimized systems specifically optimized for ocular delivery. Later non-Gag fusion and protease-free designs improved in vitro efficiency and safety but did not target RPE or photoreceptors and were unsuitable for BE/PE-specific cargo [191,192].

Lyu et al. introduced aptamer-aptamer binding protein interactions to directionally load ABE RNPs onto the HIV-1 Gag, providing a potential IRD delivery solution. However, in vivo retinal efficacy remained unproven, and aptamer fusion raised off-target concerns [193].

Engineered VLPs (eVLPs) based on MLV incorporated protease-cleavable linkers, nuclear export signals (NESs), and optimized structural ratios, which enhance RNP loading and ocular delivery [180]. In mouse models of retinal degeneration (e.g., *rd12*), ABE delivered via eVLPs has demonstrated therapeutically relevant editing and functional rescue with low off-target effects [180].

Subsequent efforts have focused on RPE targeting while confronting persistent challenges in editing photoreceptors. lentivirus-derived nanoparticles (LVNPs) packaging Cas9 or BE RNPs, achieved ~20% Vegfa knockout in mouse RPE cells via subretinal injection, with faster kinetics and fewer off-target effects than compared to mRNA delivery [181,182].

To address cargo adaptability, Raguram et al. used a barcoded sgRNA-directed evolution system to generate fifth-generation (v5) eVLPs bearing capsid mutations such as Q226P and C507V. These variants show a 24-fold increase in in vitro delivery potency over v4 eVLPs, a ~1.8-fold higher Cas9 protein packaging, and ~4.3-fold greater sgRNA enrichment while maintaining low toxicity [92].

In 2024, An et al. optimized RNP-packaged VLPs for prime editing. Their engineered PE-eVLPs employed the MCP-MS2 system for epegRNA co-delivery, NES repositioning, and the removal of protease cleavage sites, boosting editing efficiency 65–170-fold. Subretinal injection restored MFRP protein in rd6 mice (15% editing) and improved electroretinography (ERG) signals in *rd12* mice (7.2% *RPE65* editing) coupled with improved ERG responses, with minimal off-target effects, were confirmed via global off-target assays [194].

- Advancing VLPs for IRDs Therapy Toward Clinical Translation

While RNP-packaged VLPs have demonstrated therapeutic potential in preclinical IRD models, their clinical translation remains constrained by several unresolved barriers.

Photoreceptor targeting is the foremost challenge. Most VLP systems efficiently edit RPE cells but achieve negligible activity in photoreceptors. For instance, in *rd12* mice, eVLPs corrected ~12% of the *RPE65* mutation in RPE cells with functional rescue, yet failed to edit photoreceptors [73,180]. Even with directed evolution or ligand modifications, overcoming retinal tissue barriers and achieving photoreceptor-specific uptake remains elusive, with no system yet demonstrating consistent therapeutic-level editing.

Manufacturing and scalability are additional obstacles. Retrovirus-derived VLPs reach titers of only ~10^10^–10^11^ TU/mL, lower than AAV [195], and batch-to-batch variability in RNP loading or particle integrity hampers reproducibility [196]. Current purification methods such as density gradient centrifugation lack scalability for clinical-grade production [197].

Cargo complexity further complicates translation. Prime editors (~230 kDa) strain VLP capacity, necessitating modified capsids with larger luminal volume or RNA compaction strategies that risk reduced efficiency or increased off-target activity. Although An et al.’s PE-eVLPs represent a promising step, whether this design can be scaled to good manufacturing practice (GMP) production without compromising performance remains to be seen [194].

Safety considerations are critical, especially for pediatric IRD patients. Transient editor delivery (<24 h intracellular exposure) reduces off-target risks compared with viral DNA delivery, as shown for eVLPs [180]. Immunogenicity control also shows promise: PEG10-based SEND-VLPs caused no retinal inflammation in vivo, and engineered eVLPs with viral sequence minimization and PEGylation reduced antibody recognition, supporting potential for repeat dosing [188,198]. However, long-term safety data on delayed retinal toxicity or immune memory remain limited.

Moving toward clinical translation will require the following: (i) systematic receptor/ligand screening and capsid engineering to enable photoreceptor targeting, (ii) scalable suspension culture and chromatography-based purification for consistent GMP manufacturing, (iii) structural optimization to expand cargo capacity for large editors, and (iv) comprehensive long-term safety and immunogenicity studies. With these advances, VLPs could complement AAVs and LNPs as safe and versatile delivery systems for IRD gene editing.

##### Other Potential Nanocarriers

FDA-approved poly lactic-co-glycolic acid (PLGA) nanoparticles are biocompatible, low-toxic but with inherent negative surface charge hindering cellular uptake, and cationic lipid incorporation enhances their efficiency for Cas9 mRNA/plasmid DNA delivery [199]. For ocular use, hyaluronic acid (HA)-based nanoparticles (high biocompatibility, degradation resistance, outer retina persistence) promote intravitreal gene delivery to mouse vitreous/internal limiting membrane (ILM) when co-delivered with suramin, and HA-SA carrying GFP achieves sustained RPE expression, yet efficient photoreceptor transfection remains unachieved [200].

Cell-penetrating peptides (CPPs) facilitate the intracellular delivery of Cas9 protein/sgRNA/RNP via conjugation to avoid viral integration risks but exhibit low in vivo efficiency and high variability [201]. They have advantages for retinal base/prime editing (transmembrane transport for large RNPs, retinal ligand-enhanced targeting, good biocompatibility) yet face challenges like lower efficiency than that of viral vectors, difficulty penetrating retinal barriers, and poor endosomal escape, with no gene editing examples for IRDs [202,203]. Without targeted refinement, CPPs cannot compete with viral/other non-viral vectors for IRD therapy.

Exosomes (endogenous nanovesicles with immune inertness, barrier-penetrating ability) deliver gene editors to retinal cells, enable targeted RPE/photoreceptor delivery via surface modification, carry neurotrophic factors for photoreceptor protection, and reduce off-target effects [204,205,206]. They are hindered by complex/costly production/purification, low cargo loading efficiency/stability, suboptimal retinal targeting, and unproven long-term safety/efficacy, with no gene editing examples and most IRD-related applications remaining theoretical [207,208].

Extracellular contractile injection systems (eCISs) are biological nanomachines from bacteria and archaea, with nanosyringe-like complexes using needle-shaped structures to penetrate cell membranes and deliver payloads e.g., protein-based gene-editing tools, and the photorhabdus virulence cassette (PVC) is a well-characterized eCIS subtype [209]. The engineered SPEAR system derived from PVC enables the delivery of pre-assembled Cas9-gRNA ribonucleoproteins, featuring modular in vitro assembly and enhanced targeting via engineered Pvc13 tail fiber protein that uses universal conjugation domains (SpyTag and SNAP-tag) to attach full-length antibodies for cell specificity [210]. Its advantages include efficient macromolecule delivery and high cell-type specificity while limitations involve unproven biosafety, unclear therapeutic durability and unvalidated scalable production [210] and for IRDs, eCIS especially SPEAR shows promise for targeted delivery to retinal cells, although further evaluation is needed.

### 3.3. Delivery Routes for Retinal Base and Prime Editing

Delivery routes critically determine how base and prime editors reach disease-relevant retinal cells. Unlike viral and nonviral carriers that focus on packaging and protection, administration methods shape the spatial distribution, safety profile, and clinical scalability of editing therapies. Importantly, these routes parallel those established in gene augmentation therapies but exhibit distinct requirements due to the nature of base/prime editors.

#### 3.3.1. Subretinal Injection

Subretinal injection, widely applied in gene augmentation therapies such as LUXTURNA, delivers editors directly to the photoreceptor–RPE interface. In base editing studies, this approach has enabled efficient correction in preclinical IRD models and was recently extended to primate retinas, where dual AAV split-intein ABEs corrected *ABCA4* mutations with high efficiency [211]. Similarly, in vivo photoreceptor base editing has rescued rhodopsin-linked degeneration in mice [74]. Advantages include high transduction efficiency in photoreceptors and RPE cells, which are critical targets in LCA and RP [212,213]. However, this method requires retinal detachment to form one or more “blebs,” and intraoperative studies have detailed how bleb number, expansion, and location affect coverage and surgical risk [214]. Complications such as detachment, hemorrhage, and injection site atrophy remain significant [215]. Compared with augmentation, base and prime editors involve larger or more complex payloads (e.g., mRNA, RNPs, LNPs), which may influence tissue spread and immune response.

#### 3.3.2. Intravitreal Injection

Intravitreal delivery offers a minimally invasive and repeatable alternative, though ocular barriers limit efficient access to the outer retina. Gene augmentation has demonstrated limited efficacy without engineered capsids or enhancers [212]. For base editing, strategies including LNPs and cell-penetrating peptides are under evaluation to enhance retinal penetration. Recent work shows that LNPs can deliver mRNA efficiently to retinal cells ex vivo and in vivo, but distribution to photoreceptors remains suboptimal without chemical modification [216]. This route may be particularly suitable for diseases targeting inner retinal layers, such as ganglion cell–related disorders. Nonetheless, penetration efficiency, inflammation risk, and repeat-dosing safety remain key translational challenges [217].

#### 3.3.3. Suprachoroidal Injection

Suprachoroidal injection, an emerging method in gene therapy, provides posterior segment access via microneedle systems [218,219]. For base and prime editing, this route offers a balance between invasiveness and retinal coverage while reducing trauma compared to subretinal delivery. Recent trials suggest that it provides better outer retinal delivery than intravitreal injection, with enhanced targeting of photoreceptors and RPE cells [220]. However, this approach is associated with increased inflammatory risk [220]. For pediatric IRD applications, optimizing microneedle design like adjustments in needle length and bevel angle can improve delivery accuracy while minimizing tissue trauma.

#### 3.3.4. Comparative Considerations with Gene Augmentation

While subretinal, intravitreal, and suprachoroidal routes are shared across augmentation and editing strategies, the payload differences carry major implications. Augmentation uses DNA packaged in AAV vectors, often achieving therapeutic benefit with partial retinal coverage. In contrast, base and prime editing require sufficient delivery to target cells in situ, raising questions about coverage uniformity in larger human retinas and the safety of multiple injections. Clinical experience from augmentation shows that 1–2 blebs are typically created per eye, but extrapolating this to editors requires attention to distribution and tissue trauma [214]. Furthermore, immune responses differ: augmentation is limited by pre-existing capsid immunity, while editing involves the innate sensing of mRNA/protein components and localized inflammatory reactions [221]. Because gene therapy may require repeat dosing, strategies to mitigate ocular inflammation and maintain delivery efficacy are under active development [222]. Addressing these delivery-route-specific issues like bleb number, dosing strategies, inflammation management, and re-dosing feasibility, will be critical for the clinical translation of base and prime editing in IRDs.

## 4. Preclinical Efficacy and Safety

### 4.1. Proof-of-Concept Studies in Preclinical Mouse Models

The therapeutic potential of base and prime editing for IRDs was first validated in various mouse models. To establish translational feasibility, preclinical studies asked whether editors can be delivered to retinal cells in vivo, achieve therapeutically meaningful correction, and translate into durable functional rescue with acceptable safety. Here, we summarize the efficiency and safety outcomes demonstrated by BE and PE in IRD mouse models, respectively (Table 3).

#### 4.1.1. Base Editing

ABEs, with their ability to efficiently mediate A·T to G·C base conversions, have emerged as a powerful tool for correcting common point mutations in IRDs. This was first demonstrated in the *rd12* mouse model of LCA. Suh et al., utilized a lentivirus-delivered ABEmax to efficiently correct the *RPE65* nonsense mutation (c.130C > T, p.R44X) in RPE cells, achieving an average on-target editing rate of 15.95% at the gDNA level. This correction successfully restored elecexpression and retinoid isomerase activity, leading to improved ERG a- and b-wave amplitudes, optomotor responses (OMRs), visually evoked potentials (VEPs), and single V1 neuron activity [146]. Similarly, Choi et al. employed an NG-ABEmax with broader PAM compatibility to achieve higher levels of transcript correction. They reported a mean A-to-G correction rate of 54% at the cDNA level, of which 27% was precise editing. This correction translated into increased cone survival, as well as enhanced photopic ERG responses and VEPs that were sustained for at least six months [68].

In mouse models of RP, ABEs have also shown remarkable efficacy. For instance, Wu et al. successfully corrected the *PDE6B* missense mutation (c.1678C > T, p.R560C) in *rd10* mice by delivering a SpRY-ABE8e, which has a less restrictive PAM, via a dual AAV5 system. They achieved a 34.07% on-target editing rate, which resulted in the preservation of photoreceptor morphology, increased ERG amplitudes, and improved vision-guided behavior. This study further expanded the range of editable genomic sites [65].

A significant advancement has been the application of base editing to dominant inherited diseases, a challenge for conventional gene augmentation therapies like LUXTURNA. The work of Hu et al. highlights the unique advantage of BE in this context. Targeting an autosomal dominant RP (adRP) caused by a dominant-negative *RHO* mutation (p.Q344ter), they used a dual AAV8 system to deliver ABE8e. The treatment effectively preserved the outer nuclear layer (ONL) thickness, improved scotopic and photopic ERG responses, and enhanced visual acuity, thereby offering a promising therapeutic avenue for dominant IRDs [75].

Regarding safety assessments, preclinical studies of BE therapy have shown consistent results: the vast majority of studies have not detected significant off-target editing at the whole-genome level. Moreover, the subretinal injection route effectively confines editing to the eye, preventing edits in extraocular tissues [71,211,223]. Although bystander edits were detected in most studies, their frequency was lower than that of the on-target editing, and some bystander edits have been shown to be functionally silent, not affecting allele function [74,75,178].

#### 4.1.2. Prime Editing

Although base editors exhibit high efficiency and broad prospects for correcting point mutations, their application is limited to specific types of base transitions. To overcome this limitation and move towards the goal of correcting “any mutation”, PE was developed. PE can theoretically repair all types of point mutations, as well as small insertions and deletions, without the concern of bystander edits.

In the *rd12* mouse model of LCA2, Jang et al. used a dual AAV2 system to deliver a PE2 editor, achieving a 7.7% editing rate at the gDNA level. This intervention effectively rescued scotopic ERG responses and increased spatial frequency thresholds in the optomotor response test [224]. Following this, She et al. enhanced the editing efficiency to 11.4% using a dual AAV8-split PE3 system, which slowed cone degeneration and rescued cone morphology. Compared to untreated eyes, eyes treated with the dual AAV8-split PE3 system showed higher scotopic and photopic ERG a- and b-wave amplitudes. Furthermore, treated *rd12* mice spent less time in the unsafe zone during the visual cliff test [78].

In RP models, the study by Liu et al. provided direct evidence for PE’s advantages. They compared the efficacy of CBE and PE in *PDE6A* mutant mice and found that while CBE achieved a 23.8% editing rate, it was accompanied by high rates of bystander edits (13.6%) and indels (5.5%). In contrast, PE achieved a similar editing efficiency (21.5%) but produced almost no bystander edits and had a very low indel rate (0.9%) [223]. Further expanding PE’s utility, Qin et al. fused a PE with the SpCas9 variant SpRY to create the nearly PAM-less PESpRY. In retinal cells of *rd10* mice, this system achieved a 40.86% editing efficiency, leading to photoreceptor preservation that was sustainable for at least seven months, increased a- and b-wave amplitudes in the ERG, and higher visual acuity in the optokinetic tracking response (OKR) test. This strategy provides a replicable path for editing other pathogenic IRD genes that lack a suitable PAM [77]. Furthermore, Fu et al. targeted the *PDE6B* nonsense mutation c.1041C > A (p.Y347X) using an engineered pegRNA (epegRNA) that contains an additional 3′ RNA structural motif to prevent degradation. Combined with a truncated reverse transcriptase (RTΔRnH), this system corrected the nonsense mutation at a rate of 26.47% at the gDNA level. This not only restored approximately 39% of *PDE6B* protein expression and preserved photoreceptors but also significantly improved ERG responses and enhanced performance in the passive avoidance light–dark transition test and the active avoidance shuttle box learning test. These results provide a compelling preclinical basis for rescuing vision loss from genetic mutations in vivo using PE [71].

In terms of safety, PE therapy demonstrates a superior profile compared to BE. As a more precise editing method, preclinical studies show that PE virtually eliminates the potential risks associated with bystander edits, with most studies detecting only rare, if any, substitutions and indels. However, some researchers have noted that the dual AAV delivery system and subretinal injection method may, in some cases, lead to reduced ERG amplitudes or the thinning of the photoreceptor layer [69,77].

### 4.2. Preclinical Models with Higher Clinical Relevance

Data from mouse models alone are insufficient for proceeding to clinical trials. Establishing a comprehensive preclinical evidence chain, spanning from in vitro human-derived models to in vivo large animal models, is paramount for assessing the efficacy and safety of gene editing therapies prior to clinical translation. A prime example is the study by Muller et al. on the *ABCA4* (c.5882G > A, p.Gly1961Glu) mutation in Stargardt disease. The research group first screened and optimized their editing strategy in vitro using retinal organoids and human retinal explants. This led to the development of a split-intein-mediated ABE system, designated AAV5-v2-SABE1. This system achieved average editing rates of 34% in cone cells and 25% in rod cells within human retinal explants. In subsequent in vivo experiments, they not only validated this strategy in humanized mice but also, for the first time, achieved editing efficiencies as high as 75% in cone cells and 87% in the RPE of the macular region in NHPs. Alongside its high editing efficiency, AAV5-v2-SABE1 demonstrated a favorable safety profile. The deep sequencing of genome-wide candidate sites in human explants revealed no significant off-target editing. Notably, because the p.Gly1961Glu variant did not manifest a clear pathological phenotype in the mouse and human models employed, and because NHPs were wild type, functional vision outcomes could not be directly assessed. Nevertheless, given prior estimates that restoring “meaningful vision” may require at least ≥12.5% cone rescue, the observed editing levels indicate a strong potential for clinical translation [211].

### 4.3. Emerging Strategies and Key Considerations for Enhancing Efficacy and Safety

The current mainstream strategy for treating IRDs often involves DNA editing delivered via dual AAV vectors. However, this approach faces significant challenges, including the limited packaging capacity and the inherent immunogenicity of AAVs, as well as the potential oncogenic risks associated with permanent DNA alterations. Consequently, researchers are actively exploring alternative editing modalities and delivery strategies.

To address these challenges, Fry and colleagues turned their attention to RNA base editing. Unlike DNA editing, RNA editing does not alter the genomic sequence and is transient and reversible [225]. Furthermore, it occurs only in cells expressing the target gene, offering unique safety advantages for clinical translation. Through the subretinal injection of an AAV-delivered dPspCas13b-ADAR2 editor, Fry et al. successfully edited the pathogenic stop codon UAG to UIG (read as tryptophan) in the mouse retina, restoring usherin protein expression and its correct localization to the connecting cilium [69]. This work laid the foundation for transcriptome-targeting gene therapies for IRDs.

On the delivery front, non-viral systems are gaining traction, Kabra et al. employed non-viral, RPE-targeting silica nanocapsules (SNCs) to deliver ABE mRNA in a mouse model of LCA16. This approach achieved an editing efficiency of 10–17% and led to significant improvements in ERG responses. Compared to AAV and lentiviral vectors, SNCs did not significantly upregulate inflammatory markers like CD8 and Iba1 in RPE cells in vitro [178]. Although in vivo evidence is still needed, these findings suggest a favorable biocompatibility and low immunogenic profile for SNCs. Another promising transient delivery strategy involves engineered virus-like particles (eVLPs), which can mitigate the risks of genomic integration. Fu et al. delivered PE2 via eVLPs in *rd1* mice, which preserved the outer nuclear layer (ONL) structure. However, the therapeutic effect was less robust than that achieved with AAV2-delivered PE2, highlighting the ongoing need for engineering strategies to boost the efficiency of non-viral delivery systems [71]. Collectively, these studies present innovative avenues to bypass AAV-associated risks.

In addition to the choice of editing modality and delivery strategy, the administered dose is another critical factor that influences both efficacy and safety. While higher doses can enhance editing efficiency within a certain range, they are also associated with a greater risk of adverse effects. For example, She et al. reported that a very high AAV dose (3 × 10^10^ GC/eye) resulted in a significant reduction in ERG a- and b-wave amplitudes [78]. Similarly, Fry et al. observed the significant thinning of the photoreceptor layer in the injection area following subretinal AAV administration. Notably, their study further identified the dose of the gRNA-expressing vector as a key determinant of toxicity in a dual-vector system [69]. These findings underscore the necessity of dose optimization in future clinical applications to strike a balance between therapeutic efficacy and dose-dependent toxicity. Furthermore, they highlight the value of developing single-vector systems or more efficient constructs to reduce the overall viral load required for treatment.

It is worth noting that for both base and prime editing, multiple studies have highlighted that the therapeutic window is critical for efficacy. For instance, in mouse models of *RHO*-associated RP, treatment administered after significant photoreceptor degeneration (e.g., after postnatal day 21–28) yielded no significant histological or functional improvement [74,75]. Similarly, for *PDE6A*-associated RP, intervention at P14 was less effective than at P0–P3, reinforcing the necessity of treatment while the retinal structure remains largely intact [223]. This provides a key insight for future clinical translation: early diagnosis and treatment are crucial for determining long-term outcomes.

In conclusion, these rigorous preclinical data systematically demonstrate the efficacy of base and prime editing in various IRD models and have established a preliminary safety framework for their in vivo application.

## 5. Preclinical-to-Clinical Translation

BE and PE offer novel directions for curing IRDs, but translating preclinical success into clinical application remains challenging. The clinical development of BE/PE for IRDs is still at an early stage and no large-scale trials have yet been launched. Current work therefore builds on design principles, safety data, and efficacy standards established by mature IRD gene therapies, including gene augmentation therapy LUXTURNA for *RPE65*-associated LCA2, CRISPR/Cas9 therapy for *CEP290*-associated LCA10, AAV-mediated therapy for choroideremia (CHM), and phase I/II/III trials for XLRP. These therapies have established systematic insights in editor/therapeutic gene targeting design, vector optimization, injection protocols, and dosage control, which can guide BE/PE trial design and reduce exploratory risks [226].

Mature therapies validate prioritizing high-frequency mutations and using narrow editing windows to lower off-target risks. For example, CRISPR/Cas9 therapy EDIT-101 targets a 20 bp window around the *CEP290* mutation in LCA10. Its phase I/II trial (NCT03872479) showed no definite off-target effects and nearly 50% editing efficiency in human retinal organoids [226,227]. Mature therapies also emphasize matching retina-specific promoters: LUXTURNA uses the CAG/CBA promoter for RPE-specific expression, and EDIT-101 uses the GRK1 promoter to target photoreceptors, which helps minimize off-target expression [228].

AAV is the main vector in IRD gene therapy, accounting for 87% of vectors according to a systematic review and meta-analysis [229]. Clinical experience includes AAV2/8 for RPE targeting (e.g., in CHM phase II trials [230] and Bietti crystalline dystrophy (BCD) clinical studies [231]), engineered AAV2tYF for inner retinal transduction [232,233], and dual AAV split strategies (e.g., in the XIRIUS trial for *RPGR* gene supplementation [234]). AAV immunogenicity is managed via high-purity manufacturing, perioperative corticosteroids, and neutralizing antibody (NAb) screening, as used in LUXTURNA trials [235].

For injection routes and dosage, subretinal injection (the gold standard for outer retina) can reference LUXTURNA trials [235], and intravitreal injection (for inner retina) can reference XLRS trials [236]. LUXTURNA’s 1.5 × 10^11^ vg/eye dose, which balances efficacy and safety, serves as a reference for BE/PE, with adjustments based on editing efficiency [237,238].

BE/PE need to reference these mature experiences: they should prioritize high-frequency mutations, combine retina-specific promoters to avoid ectopic expression, adopt dual AAV strategies for PE delivery if needed, and draw on mature therapies’ immunological management methods. Dosage should reference LUXTURNA and adjust based on editing efficiency [228,235,238].

Detailed protocol components, such as subject selection criteria, still require customization tailored to BE and PE. Notably, defining clinical endpoints is critical for assessing the efficacy and safety of BE and PE, as existing endpoint systems validated in IRD gene therapy trials have established a comprehensive verification chain for clinical efficacy and safety evaluation [233]. The following section will discuss this in detail.

### 5.1. Functional Assessment and Clinical Endpoint Selection

The design of clinical endpoints for BE/PE trials must reflect both IRD pathophysiology (progressive photoreceptor loss, macular atrophy) and the technical features of precise mutation correction. Traditional visual assessments, which emphasize photopic function, are insufficient for advanced IRDs. Gene therapy trials for IRDs have already established a multidimensional endpoint framework covering functional improvement, structural preservation, quality-of-life impact, and safety, which can be adapted for BE/PE.

Functional endpoints directly indicate photoreceptor or RPE recovery and must match IRD subtype impairments. LCA10 patients present early rod-cone dysfunction, while XLRP shows progressive visual field loss. Best-corrected visual acuity (BCVA) via ETDRS charts remains the gold standard for mid-to-late-stage IRDs, with a meaningful improvement defined as ≥15 letters. In *CYP4V2*-related IRDs, average BCVA gain at 365 days was only about 11 letters [239], prompting alternative metrics. Low-luminance visual acuity (LLVA) better captures subtle recovery in early-stage patients, often preceding BCVA improvements. In an XLRP trial, 27% of treated eyes showed LLVA gains ≥15 letters at 12 months [240], demonstrating sensitivity for early efficacy [232].

Microperimetry (MP) evaluates macular sensitivity in patients with poor central vision or fixation and a 7 dB increase indicates functional recovery. In the *RPGR*-XLRP Phase 2/3 XIRIUS, MP showed high variability in patients with fixation deficits, contributing to trial discontinuation [241]. The multi-luminance mobility test (MLMT) designed for low-vision IRD patients assesses navigation ability under varying illumination levels. In a LUXTURNA trial, 70% of subjects passed the 1 lux MLMT at 1 year demonstrating significant functional vision gains [242]. MLMT further quantifies real-world navigation performance across illumination gradients from 1 lux to 400 lux closely approximating daily living activities [242].

Full-field stimulus testing (FST) distinguishes rod and cone function, unaffected by nystagmus, and is widely used. Standardized stimulus, dark adaptation, and reference values minimize cross-site variability [243]. A meta-analysis reported a 1.69 log cd·s/m^2^ improvement in blue FST post-gene therapy (95% CI 1.21–2.16, *p* < 0.00001) [244], correlating with subjective night vision improvements. Chromatic pupillometry further evaluates rod, cone, and intrinsically photosensitive retinal ganglion cell pathways, providing objective metrics even in ultra-low-vision patients and detecting residual function in *CEP290*-LCA cohorts [245,246].

Functional improvements require structural support. Optical coherence tomography (OCT) is the cornerstone structural tool, with ellipsoid zone width correlating with cone survival [247] and outer nuclear layer thickness reflecting photoreceptor density [248]. Fundus autofluorescence (FAF) and adaptive optics (AOs) monitor RPE health and single photoreceptor morphology, respectively. FAF-detected RPE atrophy progression often parallels functional outcomes serving as a long-term structural endpoint. In *RPGR*-XLRP trials, focal RPE atrophy appeared at subretinal injection sites at 6 months [249]. AO’s 2 μm resolution aids patient stratification by identifying surviving cones [250]. In addition, OCT angiography complements assessment by tracking microvasculature, even with fixation artifacts in BCD patients [250].

Beyond objective metrics, patient-centered outcomes offer crucial insight into how BE/PE-based treatment affect patients’ daily life. IRD patients often value improvements in dim-light vision, contrast sensitivity, mobility, reading ease, and light adaptation more than small gains in acuity alone. Patient-reported outcomes (PRO) capture quality-of-life impacts. Recently, the ViSIO-PRO/ObsRO instruments were developed specifically for RP/LCA to more comprehensively capture visual symptoms and vision-dependent activity limitations, pointing toward a future direction for IRD-specific PRO tools [251]. The Michigan Retinal Degeneration Questionnaire (MRDQ) covers seven visual domains, differentiates rod-cone versus macular dystrophies, and correlates with generic visual function scales [252]. Clinical studies in RPGR-associated retinal degeneration have begun correlating structural/functional clinician-reported outcomes with PRO scores, underscoring that anatomic rescue does not always translate to subjectively meaningful benefit for patients [253]. Nevertheless, PRO collection poses challenges: recall bias, response shifts, floor/ceiling effects, and measurement error can confound interpretation. To mitigate this, BE/PE trials should embed PRO quality control measures (e.g., baseline training, repeated assessments, electronic PRO platforms, blind scoring). Also, prior to full deployment, content validity, cultural adaptation, and psychometric testing should be conducted in the target IRD population.

Safety endpoints must address immune-related adverse events, surgical complications and delayed pathology. Standardization of Uveitis Nomenclature grading and ELISpot-based IFN-γ assays are used to evaluate anti-AAV T-cell responses [254]. In *RPGR*-XLRP trials, steroid-responsive vitritis and retinitis in high-dose cohorts underscore the need for dose-dependent immunological surveillance and steroid protocols [240]. Long-term anti-AAV antibody surveillance is also critical.

LUXTURNA follow-up data revealed Nab titer elevations without overt inflammation warranting routine post-op monitoring [255,256]. Delayed retinal atrophy, observed perioperatively or progressively in LUXTURNA-treated patients, necessitates longitudinal FAF and OCT tracking [257].

BE/PE-specific risks include off-target effects, requiring deep sequencing. Trials should verify targeted correction and confirm the absence of random integration. Endpoint selection should reference natural disease progression, using sensitive metrics like LLVA for early-stage disease and real-world function-focused tools like MLMT for advanced stages to address IRD heterogeneity.

### 5.2. Preclinical-to-Clinical Translation Barriers

The clinical translation of base editing and prime editing for IRDs faces three core barriers: preclinical model limitations (the most critical), challenges shared with other gene therapies, and unique hurdles tied to editing technologies. This section dissects these barriers and corresponding solutions.

#### Preclinical Model Limitations

Preclinical models consistently fail to fully replicate human IRD pathophysiology, creating gaps in predicting base editing and prime editing efficacy and safety. These limitations span anatomy, pathology, disease tempo, and functional assessments, often distorting therapeutic window evaluation.

Anatomically, murine retinas lack a macula, a critical target for diseases like Stargardt and LCA. Human iPSC-derived retinal organoids and non-human primate validation are needed for macula-specific validation [258]. Pathologically, some models require artificial pathology induction: *CYP4V3* knockout mice need high-fat diets to show ERG deficits, unlike human BCD. Humanized h-*CYP4V3* mut/mut mice better replicate lipid accumulation and RPE atrophy [247,259]. Temporally, disease progression is much faster in mice—rd9 degenerates in 3 months versus decades in humans—complicating therapeutic window extrapolation. For example, preclinical studies show *RHO*-associated RP treatment after murine phenotype onset (P21–P28) shows no benefit, and *PDE6A*-associated RP treatment at P14 is less effective than P0–P3. Rapid murine progression makes it hard to extrapolate to human intervention timelines [74,75,223]. Longer-lived models such as *XLPRA2* dogs, with 5-year rod-to-cone degeneration, allow for the long-term assessment of ONL preservation post-base editing [234], while dark-rearing *rd10* mice slows degeneration to better mimic chronicity [256].

Functional assessment gaps further weaken model reliability. Murine ERG does not correlate with human BCVA, and OCT-measured ONL changes cannot predict visual acuity improvements. BE/PE studies should therefore incorporate human-relevant endpoints: mobility tests primates, dose-response evaluations in *RHO P23H* pigs with variable mutation loads, and standardized FST parameters [237]. Additionally, *CEP290* mouse models lack nystagmus, limiting the replication of human OCT measurement errors. Future humanized gene-edited models with nystagmus may improve imaging reliability [260].

## 6. Challenges and Future Opportunities

### 6.1. Common Challenges Across Gene Therapies for IRDs

Vector immunogenicity is a primary shared concern. The prevalence of AAV neutralizing antibodies varies by serotype and region, requiring individualized screening at enrollment [261,262]. Empty AAV capsids over 2% trigger TLR9-mediated inflammation and gene therapy-associated uveitis, but this risk is mitigated by the AAVX platform with advanced purification and perioperative steroids validated in LUXTURNA trials [254,255]. CpG motif depletion in AAV DNA further reduces TLR9-driven CD8^+^ T-cell activation in murine models [254].

Insufficient transduction efficiency and tissue targeting affect multiple gene therapies. The internal limiting membrane blocks AAV access to the outer retina during intravitreal injection, but engineered capsids like AAV7m8 show superior traversal [257,263]. Subretinal injection enables precise targeting, with multi-site small-volume injection expanding vector distribution and robotic-assisted injection lowering retinal detachment risk [18,256]. Retinal-specific promoters and capsid engineering enhance targeting specificity [18,232].

Retinal physiological barriers and long-term follow-up are additional shared hurdles. The blood retinal barrier limits systemic delivery, making local injections preferred. Fluid air exchange after subretinal injection minimizes systemic leakage [254]. Nystagmus interferes with imaging for monitoring, but AOs improve accuracy [247]. Short-term follow-ups are inadequate for slow-progressing IRDs; so, a 10-year patient registry—mirroring *RPE65* therapy’s 15-year follow-up with annual FST, OCT and germline monitoring—is advised [258]. XLRP needs ≥10-year follow-ups for delayed retinal atrophy, while LCA2 requires ≥7.5 years for RPE stability [264].

Ethical and regulatory scrutiny represents a critical checkpoint in the clinical translation of BE/PE. Because these technologies directly modify genomic sequences, they are subject to especially stringent review, focusing on off-target activity, germline safety, and long-term risk monitoring. Fortunately, the regulatory experience gained from existing IRD gene therapies provides a solid framework that BE/PE research can build upon. Off-target assessment remains one of the key priorities for regulators. Although BE and PE generally show much lower off-target rates than conventional CRISPR/Cas9 systems, a genome-wide validation of editing specificity is still required. Using high-fidelity editor variants such as ABE8e or PE3 can further minimize unwanted edits. In preclinical and clinical stages, whole-exome sequencing (WES) and digital droplet PCR (ddPCR) should be applied to retinal tissues or iPSC-derived cells after treatment, maintaining an off-target frequency threshold below 0.01% [18]. Long-term safety data from viral-vector studies (such as those with AAV) indicate no vector presence in non-human primate reproductive organs, suggesting that local ocular administration helps avoid germline exposure. For future BE/PE trials, tissue-specific promoters could be used to restrict editor expression outside germ cells, while periodic monitoring of peripheral-blood vector copies would further ensure the absence of systemic dissemination [255,261]. Ethical review should also pay attention to data integrity and patient privacy. For example, unreliable FST data might misrepresent BE-induced visual improvements as off-target effects. Establishing strict FST quality standards will help filter out low-quality datasets and reduce interpretive bias [243]. Likewise, PRO tools such as MRDQ often involve sensitive psychological data. These datasets need de-identification procedures that preserve the phenotype-score link but eliminate personal identifiers. Developing IRD-specific PRO ethical guidelines for data storage and sharing will be crucial to maintaining confidentiality [252].

### 6.2. Unique Translational Challenges of BE and PE

Both BEs and PEs exceed the ~4.7–5 kb packaging capacity of conventional AAV vectors—prime editing editors are over 6 kb, and base editing editors have similarly large molecular sizes [261]. Lentiviral vectors pose insertional mutagenesis risks in retinal applications; so, preclinical studies rely on dual AAV split strategies that achieve measurable editing efficiency in murine models via in vivo reconstitution [261]. For the *RPGR* ORF15 region, purine-rich sequences reduce AAV packaging efficiency, but segmented cloning plus codon optimization (removing cryptic splice sites like GGTGAT) significantly improves this [265].

While base editing and prime editing have lower off-target rates than CRISPR/Cas9, rigorous evaluation is mandatory. High-fidelity variants (e.g., ABE8e, PE3) minimize off-target effects, supplemented by whole-exome sequencing and the ddPCR of treated retinal tissue or iPSCs—off-target thresholds are set below 0.01% [18]. This level of scrutiny is unique to editing technologies, as non-editing gene therapies do not alter endogenous gene loci.

Customized AAV purification for base editing and prime editing is costly, but the AAVX resin (compatible with 15 AAV serotypes) combined with suspension cell culture technology substantially reduces production expenses [256,261]. This scalability solution is critical for editing therapies, which require more complex vector design than non-editing approaches.

Unlike non-editing gene therapies, dose setting for base editing and prime editing must account for editing efficiency alongside safety. Trials reference LUXTURNA’s approved dose of 1.5 × 10^11^ vg/eye and adopt a stepwise escalation strategy to balance efficacy (driven by editing rate) and risk (e.g., retinal atrophy from high doses) [234,238]. Vector impurities like residual DNA that trigger inflammation are addressed with DNase treatment and prophylactic intravitreal corticosteroids [18,248].

Germline safety requires tissue-specific promoters to restrict editor expression in the germ cells and the monitoring of peripheral blood vector copies—LUXTURNA’s long-term data (no AAV in non-human primate reproductive organs) support this localized approach [255,261]. Data reliability demands stringent FST quality criteria to avoid misattributing functional changes to off-target effects [266]. Patient-reported outcomes involving psychological data need de-identification while preserving phenotype-score linkages, and pediatric protocols require parental consent plus developmentally appropriate assent [267]. These requirements are more rigorous for editing technologies due to their direct impact on the genome.

### 6.3. Preclinical-to-Clinical Translation Potential

The clinical translation of BE/PE for IRDs will depend on innovations in delivery platforms, preclinical models, and functional assessment strategies. Efficient retinal delivery remains a critical challenge: prime editors exceed single AAV capacity, and dual AAV split systems [234,264] still show limited editing efficiency in photoreceptors. Future directions include optimized viral capsids, lipid or hybrid nanoparticles, split-intein designs, and tissue-specific promoters to improve coverage, specificity, and safety. The mitigation of AAV immunogenicity and germline exposure is also essential [255,261,262].

Human retinal organoids and explants provide scalable, patient-relevant platforms for strategy optimization. For example, Muller et al. screened ABE designs in organoids and human retinal explants before developing AAV5-v2-SABE1, achieving high editing efficiency in cone, rod, and RPE cells while confirming minimal off-target effects [211]. Similarly, EDIT-101 for LCA10 reached nearly 50% editing in human organoids [227]. These systems allow for the preclinical evaluation of editor design, promoter selection, dosing, and off-target risk, complementing animal studies.

Large-animal and humanized models remain critical to bridging species differences. *XLPRA2* dogs, with slow rod-to-cone degeneration, enable the long-term assessment of outer nuclear layer preservation post-BE treatment [234,268], while h-Cyp4v3 mut/mut mice better model human Bietti crystalline dystrophy for RPE-targeted editing [247,259]. Combining these models with advanced imaging and functional readouts can improve predictions of human outcomes.

Innovations in delivery and dosing strategies will further enhance translational potential. Subretinal, intravitreal, and suprachoroidal administration, integrated with imaging-guided or robotic assistance, could maximize retinal coverage while minimizing trauma. Carrier modifications—e.g., LNPs, CPPs, or split-intein AAV systems—may increase editing efficiency and reduce inflammation.

Finally, a selection of clinically meaningful endpoints is crucial. Preclinical readouts from organoids or animal models should be paired with functional measures reflecting human disease, such as LLVA or MLMT, to better evaluate intervention timing and therapeutic impact across IRD stages [227,228].

In summary, the future potential of BE/PE therapies for IRDs relies on next-generation delivery strategies, advanced humanized and organoid models, and integrated functional assessment approaches. Together, these advances will help translate promising preclinical results into safe and effective clinical applications.

### 6.4. The Future: AI-Driven Advancements in Base and Prime Editing for IRD Therapeutics

#### 6.4.1. AI-Enhanced Editor Design

AI is systematically accelerating the research, development, and clinical translation of BE and PE for the treatment of IRDs with unprecedented depth and breadth.

AI can significantly enhance the efficiency of editor design. Experimentally determining the most efficient gRNA or pegRNA for a specific site is often time-consuming and labor-intensive. AI tools can effectively streamline this process. Machine learning models can learn from vast experimental datasets to identify key factors influencing gRNA or pegRNA efficiency, simultaneously predicting their activity and off-target effects. Tools like PRIDICT2.0 and ePRIDICT can predict pegRNA activity in prime editing to facilitate efficient design, also accounting for the influence of local chromatin context on editing efficiency [269,270]. Furthermore, the SynDesign web tool can design, evaluate, and construct precise pegRNA libraries for saturation genome editing, aiding the in-depth functional assessment of disease-associated genes and variants [271]. AI can also further improve CRISPR prime editing by reducing misfolded pegRNA interactions [272].

AI-based protein structure prediction can also be leveraged to develop superior base editors. Huang et al. utilized AlphaFold2 to perform the structural similarity clustering of deaminases, leading to the discovery of the novel deaminase Sdd, with Sdd6 maintaining high targeting specificity and editing activity while being smaller in size [273].

#### 6.4.2. AI-Guided Evolution of AAV Capsids

AI is accelerating AAV capsid engineering by guiding rational design beyond conventional mutagenesis. Church’s group first combined mutational scanning with fitness landscape mapping to uncover hidden elements such as MAAP [274]. Marques et al. applied ANN/SVM models to predict assembly-competent capsids and identified residues critical for particle formation [275]. Kelsic et al. expanded this approach, using deep learning to analyze >200,000 AAV2 variants, predicting over 110,000 productive capsids with enhanced diversity [276].

Recent tools further enable multi-trait optimization. While APPRAISE predicts peptide–receptor compatibility with AlphaFold-Multimer/ESMFold [277], Fit4Function integrates transduction and manufacturability, yielding capsids with an up to 1000-fold higher human hepatocyte transduction [278]. Together, these studies highlight AI’s potential to accelerate AAV design, though predictive reliability still depends on high-quality, diverse training data and iterative experimental validation.

#### 6.4.3. AI-Powered Efficiency and Off-Target Prediction

Several tools predicting PE efficiency have been developed, including DeepPrime for predicting 1–3 bp edits, PRIDICT focusing on 1bp substitutions and short indel events, and the deep Transformer model DTMP-Prime specifically designed for predicting pegRNA activity and PE efficiency [269,270]. Liu et al. proposed OPED, a more interpretable nucleotide language model, which assisted in designing PE strategies achieving an two-fold higher editing efficiency [279]. Concurrently, CRISPR-Analytics, a comprehensive gene editing web application tool, provides support for gene editing experimental design and analysis. Various computational tools and databases are continuously optimizing every stage of the gene editing workflow, from gRNA design and off-target prediction to screening analysis and biological validation [280,281].

Off-target effects are a major concern for the clinical translation of gene therapy. AI deep learning models can infer potential DNA off-target sites and their activity across the entire genome with high precision. Such models learn complex patterns in DNA sequences; for instance, Basset uses deep convolutional neural networks (CNNs) to learn DNA sequence functional activity for identifying genomic regulatory elements, subsequently providing a high-priority list for in vitro and in vivo validation [30,282]. Furthermore, faced with the massive, complex data generated by high-throughput off-target screening methods like GUIDE-seq and Circle-seq, AI algorithms can efficiently mine and identify statistically significant true off-target signals, greatly enhancing the reliability of safety assessments [30].

Beyond predicting and identifying off-target effects, AI can also model the risk of RNA off-target effects and DNA bystander editing potentially induced by deaminases. While BEs such as ABEs and CBEs enable single-nucleotide editing, endogenous factors influence in vivo editing outcomes. AI models can integrate endogenous factors beyond DNA sequence context to improve the accuracy of predicting base editing results [30,283]. Additionally, AI can integrate tools like SpliceAI to predict the potential impact of any on-target or off-target editing on mRNA splicing, thereby effectively mitigating the risk of gene therapy introducing new mutations [30].

#### 6.4.4. AI-Driven Personalized Therapy

For highly genetically heterogeneous diseases like IRDs, AI can rapidly analyze patient whole-genome sequencing data, accurately identify pathogenic point mutations, and predict their functional impact (e.g., on splicing, protein structure), thereby determining their suitability as targets for BE or PE [284].

Going further, AI can integrate a patient’s genetic variant profile to tailor the optimal treatment strategy for each individual: this includes selecting the most appropriate editor type (ABE or CBE), designing the most efficient and safe gRNA/pegRNA, and even predicting the optimal therapeutic dose and expected efficacy window [284]. Simultaneously, AI can analyze patient genetic and clinical data to predict the likely outcomes of different treatment options, supporting the development of personalized treatment plans [285]. Furthermore, AI-driven bioinformatics is transforming genomics, gene editing, and personalized medicine by analyzing large-scale genomic data to precisely identify disease-associated variants and accelerate drug discovery [284].

## 7. Conclusions

BE and PE provide precise therapeutic options for IRDs. They address the limitations of traditional gene augmentation and silencing by directly correcting pathogenic mutations, covering most point mutations and small insertions or deletions. Innovations in delivery systems, including optimized viral and non-viral carriers, have partially overcome barriers related to cargo capacity and targeting. These advancements have achieved the restoration of visual function and the preservation of retinal structure in preclinical models, with controllable safety. However, core challenges remain, such as insufficient delivery efficiency especially for photoreceptors; discrepancies between preclinical and clinical models; vector immunogenicity; and scalable production of editing tools. Future progress will depend on the AI-assisted optimization of editors and delivery designs, as well as rigorous clinical trials, to translate these technologies into curative therapies for IRDs.

## 8. Method of Literature Search

A comprehensive literature search was conducted to collect studies related to base and prime editing technologies and their applications in IRDs. Major databases including MEDLINE (PubMed edition), EMBASE, Scopus, BIOSIS Previews, Web of Science, ClinicalTrials.gov, and the World Health Organization’s International Clinical Trials Registration Platform were searched for publications from January 2015 to October 2025, with earlier foundational studies also being included when necessary to provide historical and mechanistic context. The search focused on publications addressing the molecular design and optimization of base and prime editors, and delivery strategies for retinal gene therapy, including viral and nonviral systems, as well as preclinical efficacy, safety assessment, and translational challenges. Additional emphasis was placed on studies exploring the emerging use of artificial intelligence for editor design, efficiency prediction, and personalized therapy development. Reference lists of key reviews and experimental studies were manually screened to identify additional relevant research. Only peer-reviewed English-language publications presenting original data or comprehensive analyses were included, while conference abstracts, non-English articles, and duplicate records were excluded. The initial search retrieved more than 3000 records, of which 285 publications were included based on relevance and data integrity.

## Figures and Tables

**Table 1:** Comparison of BE and PE.

	Base Editing	Prime Editing
Components	nCas9/dCas9-DNA deaminases (cytidine/adenosine deaminases)	nCas9–reverse transcriptase + pegRNA
Editing scope	Specific base conversions (C→T, T→C, A→G, G→A)	All 12 single-base substitutions; small insertions and deletions
Advantages	High editing efficiency (generally increased efficiency in non-dividing cells); simple design	Broad editable scope; high precision (minimal bystander editing); less constrained by PAM
Disadvantages	Narrow editing scope; fixed editing window with limited flexibility DNA/RNA off-targets; bystander editing	Lower editing efficiency in photoreceptors due to payload size; complex design; byproducts (unedited/partially edited); large payload, challenging delivery

**Table 2:** Comparison of delivery methods.

	AAV	LV	AdV	LNPs	Polymer-Based Nanocarriers	Inorganic Nanoparticles	Exosomes	VLPs	Electroporation
Payload Capacity	~4.7 kb	~10 kb	~30 kb	flexible	flexible	flexible	flexible	flexible	flexible
Cargo Type	DNA	DNA	DNA	RNA	RNA, RNP, DNA	RNP	RNP, RNA	RNA, RNP	RNP, RNA, DNA
Key Advantages	Non-integrating;	Efficient delivery to non-dividing cells	Non-integrating; large payload; strong expression	Biocompatibility; easy to engineer; Mature manufacturing and scale-up; scalability for GMP production	Customizable targeting	Customizable functionality	Low immunogenicity; barrier-penetrating ability	Natural viral properties (efficient intracellular delivery, endosomal escape, tissue targeting via envelope glycoproteins/ligands); transient expression window and low off-target risk; scalability for GMP production	High efficiency; strong temporal control; easy to use
Key Limitations	Pre-existing neutralizing antibodies common and redosing challenges; payload size limited; off-target risks	narrower cell targeting than AAV; integration risks	Strong immunogenicity and redosing challenges; Complex manufacturing	non-specific targeting, toxicity control; Poor stability	low transfection efficiency; toxicity control	Clearance issues (low biodegradability and accumulation risk); toxicity control; non-specific targeting; low transfection efficiency	Variable loading efficiency; purification and potency consistency challenges	Stability/consistency challenges; complex manufacturing	Impacts cell viability; not feasible in vivo

**Table 3:** Preclinical trials of BE/PE.

Study ID	Disease	Mutation	Editor	Delivery	Animal Models	Average Editing Efficiency	Structural/Functional Improvement	Safety
Muller et al., 2025 [211]	Stargardt disease	*ABCA4* c.5882G > A	ABE8.5m	Dual AAV9-PHP.eB, subretinal injection (mice); dual AAV5, subretinal injection (non-human primates, NHPs)	Mouse: *ABCA4^hu1961E/ms1961G^* NHP: cynomolgus macaques	AAV5-v2-SABE1,NHPs: 75% of cones, 87% of RPE cells	Not Applicable	No significant off-target detected in human explants; editing confined to injected eye; bystander editing: A8 (silent edit)
Hu et al., 2025 [75]	adRP	*RHO* p.Q344ter, p.T17M	ABE7.10 ABE8e	Dual AAV8, subretinal injection	*RHO^Q344ter/+^* and *RHO^T17M/+^* knock-in mice	ABE8e, *Rho^Q344ter/+^* mice, P15–P21: 15.29% at 2 months; 5.56% at P300 P29–P35: 3.18% at 2 months; 3.00% at P300	P15–21: preserved ONL thickness and scotopic and photopic ERG responses P29–35: no significant improvement	Off-target efficiency (ABE8e, *Rho^Q344ter/+^*): P15–21, 6.32%, P29-35, 1.72%
Kabra et al., 2023 [178]	LCA16	*KCNJ13* c.158G > A	ABE8e	GSH-responsive silica nanocapsules (SNC), subretinal injection	*KCNJ13^W53X/+^* knock-in mice; *KCNJ13^W53X/+ΔR^* mice (for functional readouts)	16.8% on-target	ERG c-wave recovered; OCT shows preserved retinal structure	No significant off-target, indels low; bystanders rare and mostly silent/low-frequency missense; SNC did not upregulate CD8/Iba1 versus viral vectors
Wu et al., 2023 [65]	arRP	*PDE6B* c.1678 C > T	SpRY-ABE8e	Dual AAV5, subretinal injection	*rd10* mice	cDNA: target-only 34.07%	ONL, rod OS length, scotopic/photopic ERG responses, cone structure partially preserved; water-maze success reached 100%	No significant off-target, bystander-only 1.27%; indels 0.72% histology unremarkable aside from injection artifacts (excluded)
Choi et al., 2022 [68]	LCA2	*RPE65* c.130C > T	NG-ABEmax	Single-vector lentivirus, subretinal injection.	*rd12* mice; cone-dominant *rd12^Gnat1−/−^* mice (for functional readouts)	cDNA: 54%; precise correction (no bystanders) 27%	Scotopic /photopic ERG responses, cone function and numbers preserved to 6 months VEPs present but attenuated/delayed; single-neuron responses increased.	No significant off-target; cDNA bystanders mainly at A8 (21%) and A3 (8%); indels low histology unremarkable;
Suh et al., 2020 [146]	LCA2	*RPE65* c.130C > T	ABEmax	Single-vector lentivirus, subretinal injection.	*rd12* mice	gDNA target-only: sgRNA-A5 15.95%; sgRNA-A6 5.22%;	Visual cycle (11-cis-retinal, all-trans-retinyl esters) partially reconstructed; function of the retina and entire visual pathway (ERG, OMR, VEP, V1 neurons) partially recovered	No significant off-target; indels low (A5 0.48%; A6 0.16%); bystanders mainly at T5 for A5;
Levy et al., 2020 [73]	Not a disease-correction study for the eye	None (test locus *DNMT1* to quantify in vivo efficiency)	CBE3.9max ABEmax	Dual AAV-PHP.B/Anc80, subretinal injection	*RHO-Cre*; *Ai9* mice	PHP.B + CBE: 48%in transduced rods; Anc80 + ABE: 37% in transduced rods	Not Applicable	ABE: very low indels in retinal cells. CBE: substantial indels in retina; base edit/indel ratio ~2:1 to 1:1; indels up to 34%. * no retina-specific genome-wide off-target profiling reported
Liu et al., 2024 [223]	arRP	*PDE6A* c.2009A > G	CBE4max-NG; PEmax	In vivo plasmid electroporation at P0 (subretinal + pulses); dual AAV2.NN, subretinal injection	*PDE6A* mice	CBE, electrop: 23.8% PE, electrop: 21.5% Dual AAV-PE: 9.4%	OS, ONL thickness, ERG, OKR improved; P14 injection effective but weaker than P0–P3 injection	CBE, electrop: bystander 13.6%, indels 5.5%; PE, electrop: bystander 0%, indels: 0.9%; Dual AAV-PE: bystander 0%, indels 0.4% No significant off-target (CBE, AAV-PE); Editing confined to injected eye
Du et al., 2024 [74]	arRP	*RHO* c.448G > A	SpCas9-ABEmax	Dual AAV2/8, subretinal injection	Homozygous *RHO*-E150K mice	P21 injection, cDNA: total 18.2%, precise 11.9%	P21 injection: no ERG rescue P15 injection: ERG improved, ONL preserved (in thickest treated regions); rhodopsin expression restored	No significant off-target; No detectable increase in bystander editing or indels at on-/off-target sites
Fu et al., 2025 [71]	arRP	*PDE6B* c.1041 C > A	PE2 (RT^ΔRnH^, epegRNA)	Dual AAV2, subretinal injection; engineered VLPs	*rd1* mice	AAV2, gDNA: 26.47%	AAV2: ONL, OS preserved; ERG, PLR improved; better performance in light–dark and visual cliff tests; benefits persisted to 8 weeks. (eVLPs-PE preserved ONL but was inferior to AAV-PE)	No significant off-target; editing confined to injected eye
She et al., 2023 [78]	LCA2	*RPE65* c.130C > T	PE3	Dual AAV8, subretinal injection	*rd12* mice	Precise editing (gDNA): 3.6% (low dose), 11.4% (high dose)	Retinal structure, ERG responses preserved better performance in visual cliff tests	No detectable off-target substitutions or indels above untreated background very high dose (3 × 10^10^ GC/eye) reduced ERG amplitudes
Qin et al., 2023 [77]	arRP	*PDE6B* c.1678 C > T	PESpRY: PE2 fused to SpRY	Dual AAV8, subretinal injection	*rd10* mice	76.34% in transduced cells of retinas 40.86% in total cells of retinas	Substantial rod/cone preservation; stronger rescue with P14 vs. P21 dosing; significant ERG recovery; improved OMR, avoidance tests, and water-maze; benefits persisted to at least P240	No significant off-target editing above untreated background; indels rare (0.14%); histology unremarkable
Jang et al., 2022 [224]	LCA2	*RPE65* c.130C > T	PE2	Dual AAV2, subretinal injection	*rd12* mice	7.7% (estimated 33% within transduced regions)	ERG, optomotor thresholds improved	No detectable off-target edits at 20 candidate sites, no detectable bystander, substitutions or indels at/near on-target

## Data Availability

No new data were created or analyzed in this study.

## Abbreviations

The following abbreviations are used in this manuscript:

AAV	adeno-associated virus
ABEs	adenine base editors
AI	artificial intelligence
AO	adaptive optics
ATRA	all-trans retinoic acid
AuNPs	gold nanoparticles
AYBEs	adenine transversion base editors
BCD	Bietti crystalline dystrophy
BCVA	Best-Corrected Visual Acuity
BE	base editing
CBEs	cytosine base editors
CGBEs	C-to-G base editors
CHM	choroideremia
CNNs	convolutional neural networks
CORD	cone-rod dystrophy
CPPs	cell-penetrating peptides
ddPCR	digital droplet PCR
DOPE	1,2-dioleoyl-sn-glycero-3-phosphoethanolamine
DOTAP	1,2-dioleoyl-3-trimethylammonium-propane
DSBs	double-strand breaks
eCIS	extracellular contractile injection systems
ERG	electroretinography
FAF	fundus autofluorescence
FST	full-field stimulus testing
GMP	good manufacturing practice
gRNA	single guide RNA
HDR	homology directed repair
IRDs	inherited retinal diseases
ITRs	inverted terminal repeats
LCA	Leber congenital amaurosis
ILM	internal limiting membrane
LLVA	low-luminance visual acuity
LV	lentiviruses
mitoBEs	mitochondrial base editors
MLMT	multi-luminance mobility test
MLV	moloney murine leukemia virus
MP	microperimetry
MRDQ	Michigan retinal degeneration questionnaire
NES	nuclear export signals
NHPs	non-human primates
OCT	optical coherence tomography
OKR	optokinetic tracking response
OMRs	optomotor responses
ONL	outer nuclear layer
OS	outer segment
PAM	protospacer adjacent motif
PBS	primer binding site
PE	prime editing
PEG	polyethylene glycol
PLR	pupil light reflex
PLGA	poly lactic-co-glycolic acid
PRO	Patient-reported outcomes
PVC	photorhabdus virulence cassette
RP	retinitis pigmentosa
RPE	retinal pigment epithelial
RTT	reverse transcription template
SMOFs	surface-supported metal-organic frameworks
SNPs	silica nanoparticles
TALE	transcription activator-like effector
VEPs	visually evoked potentials
WES	whole-exome sequencing
XLRP	X-linked retinitis pigmentosa
XLRS	X-linked retinoschisis
